# A Prospective Five-Year Follow-up After peg-Interferon Plus Nucleotide Analogue Treatment or no Treatment in HBeAg Negative Chronic Hepatitis B Patients

**DOI:** 10.1016/j.jceh.2021.12.011

**Published:** 2022-01-04

**Authors:** Robin Erken, Vladimir V. Loukachov, Annikki de Niet, Louis Jansen, Femke Stelma, Jeltje T. Helder, Martine W. Peters, Hans L. Zaaijer, Neeltje A. Kootstra, Sophie B. Willemse, Hendrik W. Reesink

**Affiliations:** ∗ Amsterdam UMC, University of Amsterdam, Department of Gastroenterology and Hepatology, Amsterdam Gastroenterology Endocrinology Metabolism, Meibergdreef 9, Amsterdam, the Netherlands; † Department of Experimental Immunology, Amsterdam UMC, Location AMC, Meibergdreef 9, 1105AZ, Amsterdam, the Netherlands; ‡ Department of Gastroenterology and Hepatology, Amsterdam UMC, Location AMC, Meibergdreef 9, 1105AZ, Amsterdam, the Netherlands; § Sanquin Blood Supply Foundation, Plesmanlaan 125, Amsterdam, the Netherlands; || Department of Experimental Immunology, Amsterdam UMC, Amsterdam Infection & Immunity Institute, University of Amsterdam, Amsterdam, the Netherlands

**Keywords:** combination therapy, hepatitis B virus, inactive carrier, low viral load, functional cure, ADV, Adefovir dipivoxil, ALT, Alanine aminotransferase, CHB, Chronic hepatitis B, EOT, End of treatment, GZ, Grey zone, HBeAg, Hepatitis B e antigen, HBsAg, Hepatitis B surface antigen, HCC, Hepatocellular Carcinoma, HNCH, HBeAg-negative chronic infection, NA, Nucleot(s)ide analogue, peg-IFN, Pegylated-interferon, ROC, Receiver operating characteristic, TAF, Tenofovir alafenamide fumarateor, TDF, Tenofovir disoproxil fumarate, ULN, Upper limit of normal, UMC, University Medical Centers

## Abstract

**Background:**

Currently available treatment options for chronic hepatitis B (CHB) are not recommended for HBeAg-negative patients with a low viral load. These patients may however benefit from treatment by achieving a functional cure, defined by HBsAg-loss and undetectable HBV DNA. This study evaluated the long-term effect of combination treatment with peg-interferon-alpha-2a (peg-IFN) and adefovir or tenofovir compared to no treatment in these patients.

**Methods:**

HBeAg-negative CHB patients with HBV-DNA levels < 20,000 IU/mL (*n* = 151) were previously randomised 1:1:1 for peg-IFN 180 μg/week plus either adefovir 10 mg/day or tenofovir 245 mg/day, or no treatment and treated for 48 weeks in an open-label study. In this prospective long-term follow-up study, patients were monitored yearly up to five years after end of treatment (week 308). The primary outcome was sustained HBsAg-loss and secondary outcome the dynamics of HBsAg and HBV-DNA levels over time.

**Results:**

Of the 131 followed patients, the HBsAg-status was known for 118 patients after five-year follow-up. HBsAg-loss occurred similarly (*P* = 0.703) in all arms: 8/43 (18.6%) peg-IFN + adefovir, 4/34 (11.7%) peg-IFN + tenofovir, and 6/41 (14.6%) among the untreated patients. The time to HBsAg-loss did not differ between groups (*P* = 0.641). Low baseline HBsAg levels and genotype A were independently associated with HBsAg-loss irrespective of allocation. HBsAg and HBV-DNA levels declined similarly during follow-up in all patient groups.

**Conclusions:**

This prospective randomised controlled study showed that HBsAg-loss overtime was not influenced by treatment with a combination of nucleotide analogue and Peg-IFN. Low baseline HBsAg levels can predict HBsAg-loss irrespective of treatment allocation.

Worldwide, more than 240 million people live with chronic hepatitis B (CHB) infection. These patients are at risk of developing cirrhosis and hepatocellular carcinoma (HCC), which to an overall estimated mortality of 887,000 CHB patients per year.^1^ The risk of developing hepatitis B virus (HBV)-related complications is especially preeminent in patients with elevated alanine transaminase (ALT), a high viral load and/or liver fibrosis.^2^^,^^3^ For these patients, viral suppressive therapy can reduce the risk of HBV-related complications but there is no effective curative treatment available.^3^

The majority of CHB patients,^4^ have a low viral activity (HBV-DNA < 20,000 IU/mL) and no signs of liver inflammation or fibrosis and therefore, do not meet the criteria for treatment intervention.^3^^,^^5^ The risk of cirrhosis and HCC is low in these patients, but still higher compared to uninfected individuals. A relative risk for HCC of 9.6 in nine years follow-up and a lifetime risk of 16–25% has been described,^6^^,^^7^ as well as cirrhosis in 5–6% of patients after 13 years.^8^ Furthermore, these patients are in need of medical monitoring due to the risk of viral reactivation and can still transmit the virus. A significant health gain can therefore be expected from curing this group of currently untreated patients.

An inactive state of the virus, classified as a functional cure, is currently considered the most achievable curative state for CHB.^9^ Patients who are functionally cured have a sustained (> 6 months) undetectable level of the viral protein Hepatitis B surface antigen (HBsAg) and undetectable HBV DNA.^3^^,^^9^^,^^10^ After achieving functional cure, patients are at lower risk of developing CHB-related complications and cannot infect others with HBV. Patients that do not have cirrhosis can even cease medical monitoring. Spontaneous HBsAg-loss can annually be seen in 0.05–2% untreated Hepatitis B e antigen (HBeAg) negative patients with a low viral load.^4^^,^^10^^,^^11^

Currently, nucleo(s)tide analogues (NAs) and pegylated-interferon (Peg-IFN) are the two registered treatment options for CHB. Standard of care is indefinite viral suppressive therapy with the NAs tenofovir disoproxil fumarate (TDF), tenofovir alafenamide fumarateor (TAF) or entecavir. A downside of this treatment is that it suppresses viral replication but rarely leads to HBsAg-loss. In addition to NA-therapy, treatment with monotherapy Peg-IFN for 48 weeks may lead to a sustained viral suppression and in approximately 3% to HBsAg-loss.^12^^,^^13^ Due to its unfavourable side-effects, Peg-IFN is currently mainly used as an immune modulator in combination with new treatments options under development.

Combining these two treatments was hypothesised to increase functional cure rates by simultaneously suppressing the viral replication and modulating the host immune response. Indeed, in mainly HBeAg positive patients with a high viral load, a 48 week combination treatment led to HBsAg-loss in up to 7.3–17% of patients.^12^^,^^14^^,^^15^ In these studies, low baseline HBsAg levels were related to functional cure in HBeAg negative patients. We therefore hypothesised that the rate of HBsAg-loss after Peg-IFN/nucleot(s)ide combination therapy may even be higher in patients with a low viral who also have lower HBsAg levels at baseline. In addition, these patients already have a more effective HBV specific immune response suppressing viral activity compared to CHB patients with high viral load and thus, might be more prone to achieve HBsAg-loss.

In our initial study we randomised HBeAg-negative patients with a low viral load for 48 weeks of treatment with Peg-IFN plus either ADV or TDF, or for no treatment. Adefovir is currently no longer used for long-term viral suppression due to the risk of viral resistance. It does however have the potential to improve T-cell immune function and therefore may support the immune modulating effect of Peg-IFN.^16^ Results up to 72 weeks were reported previously and showed similar rates of HBsAg-loss between all study arms.^17^ HBsAg-levels did however decline after therapy when compared to the untreated patients and remained lower compared to baseline during follow-up. In addition, off-treatment responses have been described to be more prevalent in HBeAg negative compared to HBeAg positive patients treated with Peg-IFN.^18^ This suggests a long-term effect of Peg-IFN + TDF/ADV combination treatment but has never been studied in a prospective randomised controlled trial.

The aim of this study was to prospectively evaluate the five-year rate of HBsAg-loss in HBeAg-negative CHB patients with a low viral load, randomised for treatment with Peg-IFN and adefovir, Peg-IFN and tenofovir or no treatment.

## Methods

### Initial Study

A total of 151 CHB patients were included in the previously described prospective open-label randomised controlled trial at the University Medical Centers (UMC) Amsterdam, location AMC (ClinicalTrials.gov, number NCT00973219). Patients were 18–70 years old, HBsAg-positive (> 6 months) and HBeAg-negative. Furthermore, baseline HBV-DNA levels were below 20,000 IU/mL in all patients. The main exclusion criteria were concurrent infection with hepatitis C virus, hepatitis D virus, human immunodeficiency virus; decompensated liver disease, HCC, a history of bleeding from oesophageal varices, ALT levels greater than five times the upper limit of normal (5 × ULN), or signs of liver cirrhosis (based on notes in their medical history, transient elastography (Fibroscan) measurement and/or biopsy results). Patients were either treatment naïve or had received Peg-IFN or NAs more than six months before inclusion.

### Randomisation and Masking

Patients were randomly assigned (1:1:1) to receive pegylated interferon alfa-2a (Peg-IFN, Pegasys; F Hoffman–La Roche, Basel, Switzerland) 180 μg/week in combination with either adefovir dipivoxil (ADV, Hepsera; Gilead Sciences, Foster City, CA, USA) 10 mg daily or tenofovir disoproxil fumarate (TDF, Viread; Gilead Sciences, Foster City, CA, USA) 245 mg daily, or no treatment for 48 weeks. Randomisation was stratified according to HBV genotype A, genotype B–G, or indeterminable genotype. Patients who received at least one dose of study medication or attended at least one study visit (no-treatment group) were included in the modified intention to treat analysis (*n* = 134). Liver biopsies for fibrosis grading were taken at baseline and at week 48.

### Follow-up Study

In the current long-term follow up study, patients were monitored once per year during five years after end of treatment (EOT). The long-term follow-up period for patients in the no-treatment arm, started 48 weeks after inclusion. The following parameters were documented: laboratory blood test results, physical examination, abdominal ultrasound, and transient elastography measurement. For the last visit of the long-term follow-up, a difference up to four months was accepted between planned and attended visit. Screening for HCC with a six-monthly abdominal ultrasound was performed in patients with an elevated risk for HCC, according to EASL guidelines.^3^ Patients without an indication for HCC screening received an abdominal ultrasound every 3–5 years. Patients who were lost to follow-up were actively approached and data on HBsAg status was retrieved from other centres if possible. Both the initial and the follow-up study were conducted according to the guidelines of the Declaration of Helsinki and with the principles of Good Clinical Practice, and were approved by the local ethics committee. All patients gave written informed consent.

### Laboratory Testing

Biochemical, hematological and virological laboratory analysis were carried out by the local diagnostic laboratory in accordance with good laboratory practice. ALT levels were expressed as absolute values (U/L) or relative to the ULN range (45 U/L for men and 34 U/L for women). HBV-DNA levels in plasma were determined using the COBAS TaqMan assay (F Hoffmann-La Roche, Basel, Switzerland). Qualitative detection of serum HBsAg, antibody to HBsAg (anti-HBs), HBeAg, and antibody to HBeAg (anti-HBe) was done using an enzyme immunoassay (AxSYM; Abbott Laboratories, Abbott Park, IL, USA). Quantified serum HBsAg measurement was carried out by Sanquin Diagnostics (Amsterdam, the Netherlands) using the Abbott HBsAg Architect assay (Abbott Diagnostics, Abbott Park, IL, USA). The modified Ishak scoring system was used for histological assessment of liver biopsies in the initial study.

### Outcomes

The primary outcome was the proportion of patients achieving HBsAg-loss > 6 months, up to five years after combination therapy or no treatment. Possible baseline predictors of HBsAg-loss were analysed. The secondary objective was to study the dynamics of HBsAg-, HBV-DNA- and ALT-levels over the five-year period. Furthermore, we analysed changes in fibrosis or steatosis scores, the occurrence of HCC and the persistence of long-term adverse events after treatment. Post-hoc analysis assessed baseline predictors of HBsAg-loss during follow-up and compared patients with HBV-DNA < 2000 IU/mL and normal ALT levels (HBeAg-negative chronic infection phase), with patients with HBV-DNA > 2000 IU/mL and/or elevated ALT levels at baseline.

### Statistical Snalysis

Proportions of patients with HBsAg-loss in different groups were compared using a χ^2^-test. The modified intention to-treat analysis was used in which all patients were included who received at least one dose of study drugs (treatment groups) or had at least one study visit (no treatment group). Patients from whom the qualitative HBsAg status was known at end of follow-up, were included in the analysis. Missing data due to missing patient visits were excluded from qualitative data analysis. Occurrence of HBsAg-loss over time was shown in a Kaplan Meier curve and compared between treatment groups using a log-rank test. Patients who were lost to follow-up or did not reach HBsAg-loss at end of follow-up were censored. Groups mean or medians of the biochemical, hematological, and virological laboratory tests were compared with a student's t-test, Mann Whitney-U test or an ANOVA repeated measurements test where appropriate. Predictors of HBsAg-loss were determined with a univariate and multivariate regression analysis. The accuracy of baseline HBsAg-levels to predict HBsAg-loss was assessed by means of a receiver operating characteristic (ROC) curve. Statistical analyses were performed in SPSS (IBM SPSS Statistics, version 26.0 Chicago, Illinois ll). All *P*-values below 0.05 were considered statistically significant.

## Results

### Patients

**Figure 1:**
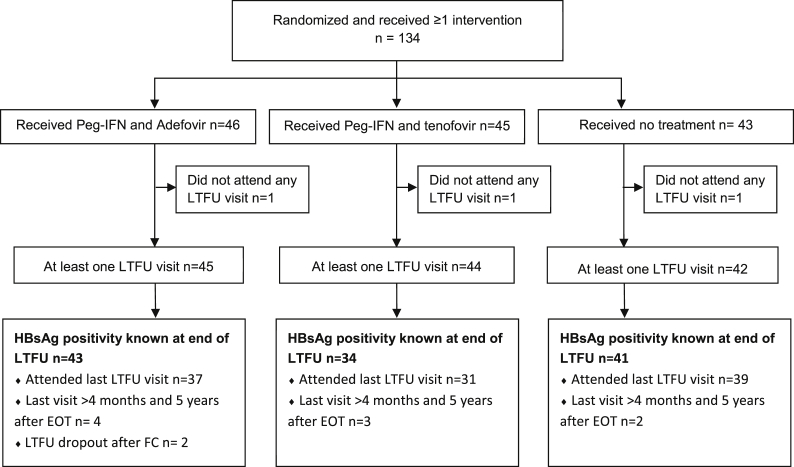
**Summarising flow diagram of inclusion and follow-up.** EOT, end of treatment; HBsAg, hepatitis B surface antigen; LTFU, long term follow-up; Peg-IFN, pegylated interferon.

In the modified intention to treat analysis, 46 patients were treated with peg-IFN + ADV, 45 with peg-IFN + TDF and 43 were untreated. At least, one visit of the long-term follow-up was attended by 131 (98%) of these patients. The primary outcome, HBsAg-positivity, was documented for 118 (88%) patients at the end of five-year follow-up (308 weeks from baseline) and they were included in our current analysis, Figure 1 and Supp. Figure 1. Distribution of patients between groups was similar: 43 (36.4%) patients in the peg-IFN + ADV group, 34 (28.8%) patients in the peg-IFN + TDF group and 41 (34.7%) patients in the untreated group. Baseline characteristics of patients who completed the long-term follow-up are shown in Table 1. For 11 out of 118 patients included in the primary analysis, the five -year follow-up visit was missing. Either the visit was conducted later than the four month time window (*n* = 9) or patients had reached HBsAg-loss and did not complete the long-term follow-up (*n* = 2). The 16 patients included in the initial study, who were lost to long-term follow-up had their last visit before the first follow-up visit (*n* = 1), at year 1 (*n* = 3), year 2 (*n* = 3), year 3 (*n* = 3), or year 4 (*n* = 6) of follow-up.

**Table 1:** Baseline Characteristics.

	Peg-IFN plus ADV (*n* = 43)	*Peg-IFN plus TDF (n = 34)*	No treatment (*n* = 41)
Age, years	45 (12)	44 (11)	42 (10)
Female sex	17 (39.5)	19 (55.9)	13 (39.0)
Ethnicity			
Caucasian	10 (23.3)	11 (32.4)	16 (39.0)
Asian	18 (41.9)	7 (20.6)	13 (31.7)
African	7 (16.3)	8 (23.5)	7 (17.1)
South American	8 (18.6)	8 (23.5)	5 (12.2)
ALT (U/L)	27 (21–42)	26 (19–31)∗	31 (22–48)
Peg-IFN naive	40 (93)	31 (91)	41 (100)
HBV genotype			
A	10 (23.3)	7 (20.6)	8 (19.5)
B	4 (9.3)	3 (8.8)	2 (4.9)
C	2 (4.7)	1 (2.9)	3 (7.3)
D	10 (23.3)	11 (32.4)	11 (26.8)
E	9 (20.9)	4 (11.8)	8 (19.5)
G	0 (0)	0 (0)	1 (2.4)
Indeterminable	8 (18.6)	8 (23.5)	8 (19.5)
HBsAg (log_10_ IU/mL)	3.18 (0.98)	3.42 (0.65)∗	3.03 (0.89)
HBV-DNA (log_10_ IU/mL)	2.64 (1.27)	2.81 (1.06)	2.76 (1.06)
HBV-DNA < 2000 IU/mL, yes	30 (69.8)	20 (58.8)	29 (70.7)
Fibroscan performed	39 (90.6)	26 (76.5)	39 (95.1)
Value kPa (median, IQR)	5.0 (1.8)∗	5.3 (1.7)	5.8 (2.0)
Liver biopsies performed	35 (81.4)∗∗	28 (82.4)∗∗	19 (46.3)
Ishak fibrosis score			
0	7 (20.0)	3 (10.7)	4 (21.0)
1	23 (65.7)	17 (60.7)	14 (73.7)
≥2	5 (14.3)	8 (28.6)	1 (5.3)
Steatosis grade			
0	22 (62.9)	17 (60.7)	13 (68.4)
1	10 (28.6)	8 (28.6)	2 (10.5)
≥2	3 (8.5)	2 (7.1)	4 (21.1)
Unknown	0 (0.0)	1 (3.6)	0 (0.0)

Data are n (%), median (IQR), or mean (SD). ADV, adefovir dipivoxil; ALT, alanine aminotransferase; HBV, hepatitis B virus; HBsAg, hepatitis B surface antigen; Peg-IFN, peg-interferon-alfa-2a; TDF, tenofovir disoproxil fumarate. ∗*P* ≤ 0.05, ∗∗*P* < 0.001 difference compared to no treatment arm.

### HBsAg-Loss

**Figure 2:**
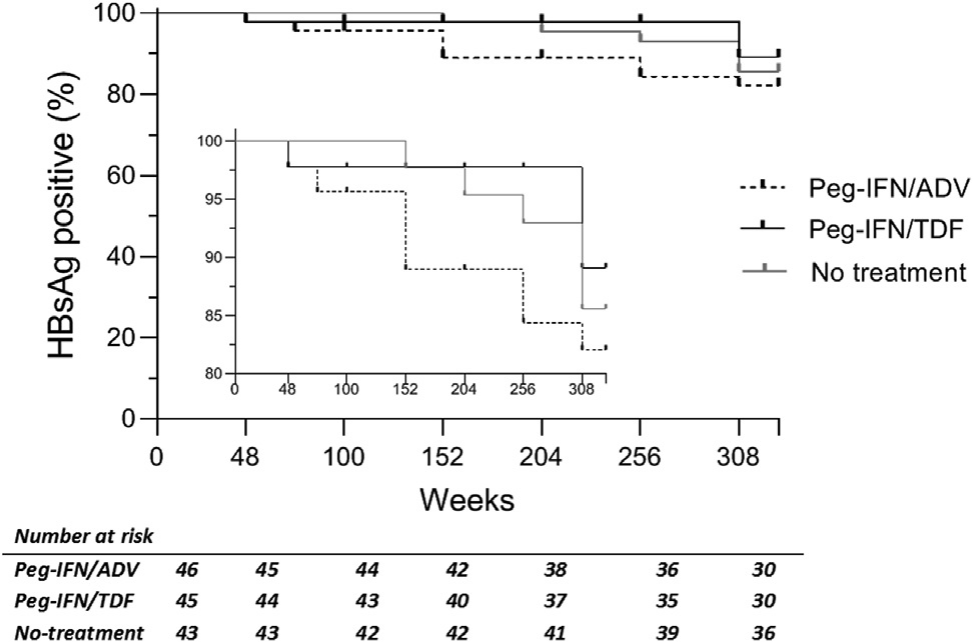
**Kaplan Meier curve of HBsAg-loss events over time.** Proportion of HBsAg-positive patients shown over time for each treatment group. The top 20% of events are displayed in the middle of the figure. HBsAg, hepatitis B surface antigen; no, no treatment arm; Peg-IFN + ADV, pegylated interferon plus adefovir; Peg-IFN + TDF, pegylated interferon plus tenofovir.

Of the 118 patients included in the analysis, HBsAg-loss was achieved in 8/43 (18.6%) patients treated with Peg-IFN + ADV, in 4/34 (11.7%) patients treated with Peg-IFN + TDF and in 6/41 (14.6%) of the untreated patients. There was no difference in the proportion of patients achieving HBsAg-loss after 5 year follow-up between groups (*P* = 0.703). In the initial study, a total of four patients achieved HBsAg-loss within 72 weeks. One of these patients was HBsAg-negative and anti-HBs positive (42.2 IU/L) at 72 weeks but converted back to an HBsAg-positive status one year after the EOT. An additional 16 patients achieved HBsAg-loss during the five-year follow-up period, Figure 2. Of one patient, the exact time of HBsAg-loss was unknown.

### Dynamics of Virology Markers

**Figure 3:**
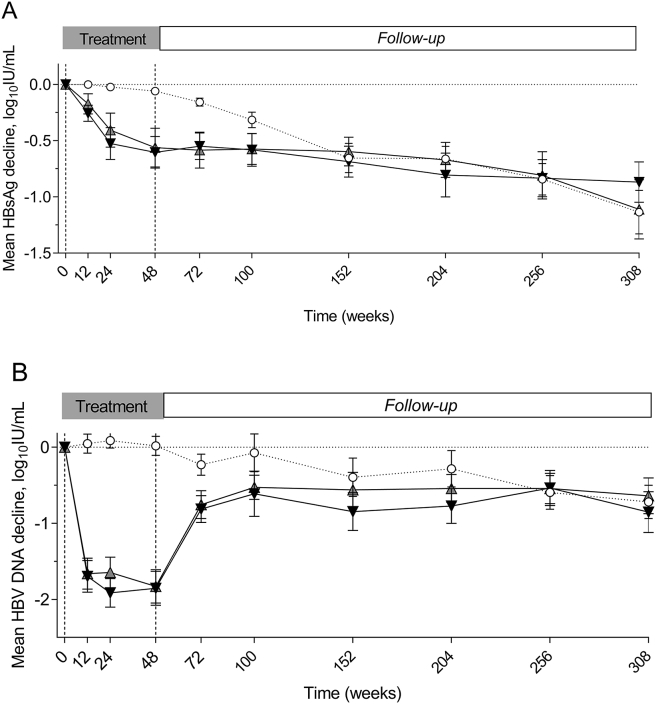
**A-B, dynamics virology marker in different treatment groups**. HBsAg (A) and HBV DNA (B) change compared to baseline, over time, standard deviation. Symbols; ▼ black downward triangle, interferon + adefovir; ▲ grey upward triangle, interferon + tenofovir; ○ circle, no-treatment group. HBsAg, hepatitis B surface antigen; HBV, hepatitis B virus.

Our secondary objective was to monitor HBsAg and HBV DNA levels during treatment and follow-up. The dynamics of these markers were similar between the two treatment groups but differed from the untreated patients (Figure 3A–B). The treatment groups combined (*n* = 77) showed a decline in HBsAg-levels up to −0.59 log_10_ IU/mL between baseline and EOT (48 weeks) compared to a decline of −0.06 log_10_ IU/mL in the untreated group. Lower (*P* = 0.035) HBsAg-levels in the treatment versus the untreated group were seen up to one year after EOT and were again similar between groups at time points thereafter. At five year follow-up, HBsAg levels had declined (*P* < 0.001) with a mean of −0.87 log_10_ IU/mL (SD, 0.94), −1.11 log_10_ IU/mL (SD, 1.34) and −1.14 log_10_ IU/mL (SD, 1.10) in the Peg-IFN + ADV, Peg-IFN + TDF and no-treatment group respectively compared to baseline. HBV DNA levels declined with maximum −1.84 log_10_ IU/mL between baseline and EOT in both treatment arms while HBV DNA levels remained similar in the untreated patients. From 1-year follow-up onwards, HBV DNA levels between al 3 groups were similar and declined (*P* < 0.001) from baseline to five year follow-up with a mean of −0.85 log_10_ IU/mL (SD, 1.58), −0.64 log_10_ IU/mL (SD, 1.31) and −0.72 log_10_ IU/mL (SD, 1.35) in the peg-IFN + ADV, peg-IFN + TDF and no-treatment group, respectively.

### Subgroup Analysis of Patients Achieving HBsAg-Loss

**Figure 4:**
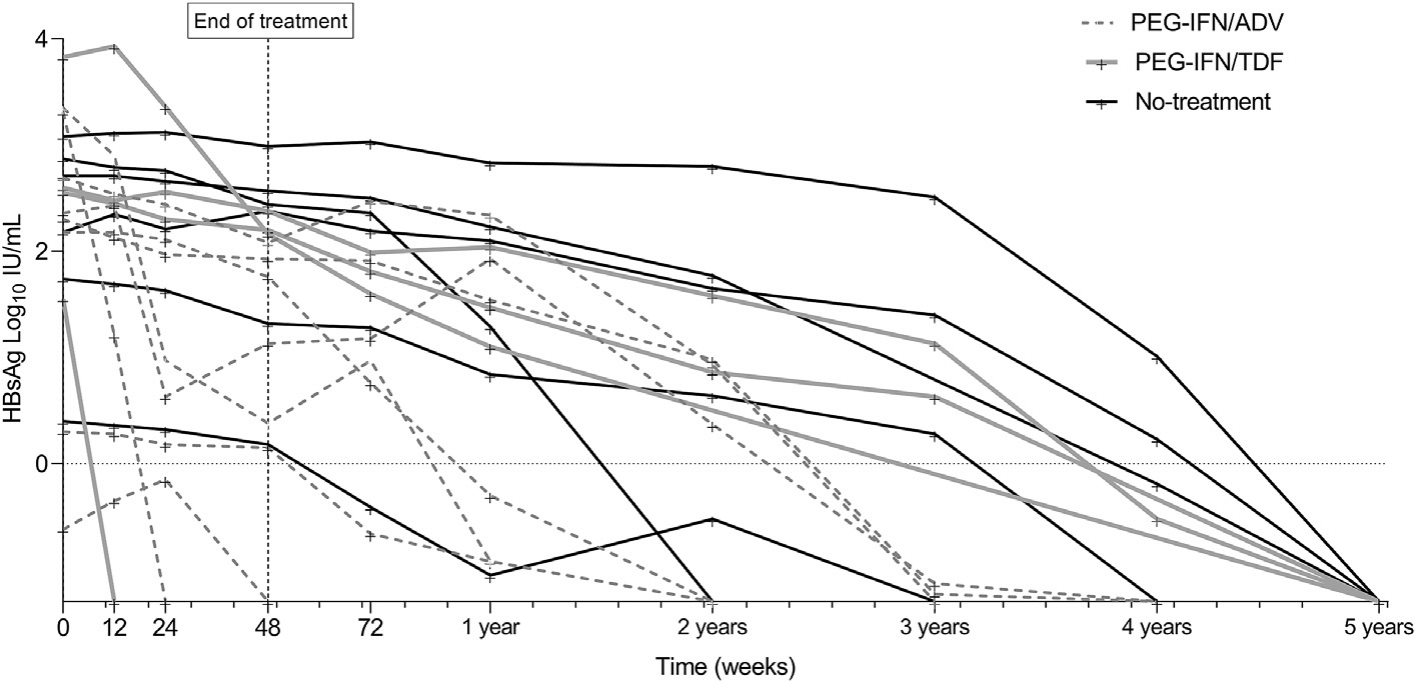
**Individual HBsAg dynamics of patients achieving FC.** HBsAg, hepatitis B surface antigen; Peg-IFN + ADV, pegylated interferon plus adefovir; Peg-IFN + TDF, pegylated interferon plus tenofovir.

Individual HBsAg dynamics for all patients achieving HBsAg-loss are shown in Figure 4. Time until HBsAg-loss was similar (*P* = 0.182) between the two treatment groups and the untreated control group: a median of 2.9 years (IQR, 2.2–4.9) in the Peg-IFN + ADV group, 5.9 years (IQR, 3.4–5.9) in the Peg-IFN + TDF group and 5.4 (IQR, 3.9–5.9) years in the untreated patient group. HBsAg levels were lower (*P* < 0.001) in patients that achieve HBsAg-loss compared to patients who did not, in all visits from baseline to the end of follow-up (Supp. Figure 1), irrespective of allocation arm. For HBV DNA, treated patients achieving HBsAg-loss had similar levels at baseline compared to patients who did not achieve HBsAg-loss. However, in the patients who lost HBsAg, a steeper HBV DNA decline (*P* < 0.05) during treatment was seen. Also, a lower rebound and further decline of HBV DNA (*P* < 0.01) after EOT was seen in these patients.

### Markers for Prediction of HBsAg-Loss

Baseline characteristics of these patients were compared to those who did not achieve HBsAg-loss after long-term follow-up for each (no)treatment arm separately (Supp. Table 1), showing that patients achieving HBsAg-loss had lower (*P* < 0.01) baseline HBsAg levels in all arms compared to patients who did not achieve HBsAg-loss. Furthermore, patients who achieved HBsAg-loss in the Peg-IFN + ADV group were younger (*P* = 0.020) with lower (*P* = 0.036) ALT levels. Patients with HBsAg-loss in the Peg-IFN + TDF group had lower (*P* = 0.048) HBV DNA levels. In patients who achieved HBsAg-loss without receiving treatment, stage 1 fibrosis and the absence of steatosis were more often seen (*P* = 0.043 and *P* = 0.041 respectively) compared to patients in this arm who did not achieve HBsAg-loss.

Since combination treatment did not have an effect on HBsAg-loss, all groups were combined in the regression analysis of baseline predictors for HBsAg-loss. Variables that were significantly associated with HBsAg-loss or showed a trend (*P* < 0.06) in a univariable regression analysis, were included in the multivariable logistic regression analysis Table 2. The multivariable regression analysis was also corrected for predefined variables such as age, sex, genotype A and treatment allocation. In this analysis, baseline HBsAg levels as well as genotype A, were found to be independent predictors of HBsAg-loss. Analysing patients with genotype A separately from patients with other or unknown genotypes showed no significant differences between these groups in terms of HBsAg-loss frequency and HBsAg decline over time (Supp. Table 2 ad Supp. Figure 2). Based on ROC analysis, a HBsAg cut-off value of 2.89 log_10_ IU/mL (AUC, 0.841) was found to identify patients who achieved HBsAg-loss before end of follow-up, with a sensitivity of 0.80 and specificity of 0.78 (Supp. Figure 3). This cut-off provides a negative predictive value of 95% and a positive predictive value of 41%.

**Table 2:** Logistic Regression Analysis of Predictors of Functional Cure.

	Univariable regression analysis			Multivariable analysis		
	B (SE)	OR (95%CI)	*P* value	B (SE)	OR (95%CI)	*P* value
Age, years	0.07 (0.03)	1.07 (1.02–1.13)	**0.010**	0.02 (0.03)	1.02 (0.95–1.09)	0.329
Female sex	−0.53 (0.54)	0.59 (0.20–1.69)	0.323	0.36 (0.69)	1.43 (0.37–5.52)	0.602
Ethnicity						
African	0.29 (0.67)	1.34 (0.34–5.27)	0.677			
Asian	−0.25 (0.57)	0.78 (0.26–2.38)	0.663			
ALT (U/L)	−0.01 (0.01)	0.99 (0.97–1.02)	0.586			
IFN naive	−0.11 (1.13)	0.90 (0.10–8.14)	0.921			
HBV genotype A	1.06 (0.55)	2.90 (0.99–8.50)	0.053	−2.25 (0.82)	0.11 (0.02–0.52)	**0.006**
Treatment						
ADF + Peg-IFN	−0.40 (0.52)	0.67 (0.24–1.86)	0.445			
TDF + Peg-IFN	0.41 (0.61)	1.50 (0.46–4.93)	0.504			
Treatment yes/no	−0.07 (0.54)	0.93 (0.32–2.69)	0.891	0.94 (0.78)	2.55 (0.55–11.77)	0.230
Baseline HBsAg (log_10_ IU/mL)	−1.57 (0.40)	0.21 (0.10–0.46)	<**0.001**	−2.21 (0.64)	0.11 (0.03–0.39)	**0.001**
Baseline HBV-DNA level (log_10_ IU/mL)	−0.52 (0.23)	0.59 (0.38–0.93)	**0.024**	0.20 (0.33)	1.23 (0.65–2.32)	0.530
HBV-DNA <2000 IU/mL, yes	1.03 (0.67)	2.81 (0.76–10.37)	0.120			
Ishak fibrosis score ≤1	−1.12 (1.08)	0.59 (0.04–2.72)	0.300			
Steatosis grade 0	0.72 (0.59)	2.05 (0.64–6.56)	0.229			

Univariable and multivariable logistic regression analysis of predictors of HBsAg-loss determined at 5-year follow-up. The multivariable regression analysis was corrected for age, sex, genotype A and treatment allocation. HBV, hepatitis B virus; HBsAg, hepatitis B surface antigen; B, regression coefficient; SE, standard error; OR, odds ratio. Significant p values <0.05 in bold.

### Subgroup Analysis of Patients in the HBeAg Negative Chronic Infection Phase

The EASL guidelines define the HBeAg-negative chronic infection (HNCH) phase as an HBeAg-negative state with HBV DNA levels ≤2000 IU/mL and normal ALT-levels, previously known as inactive carrier state.^3^ In our cohort, 72 (61%) patients met these criteria at baseline while 46 (39%) of patients fall into an indeterminate grey area between chronic infection and chronic hepatitis with HBV-DNA levels > 2000 IU/mL and/or ALT levels >1.25. These patients were classified as ‘grey zone’ (GZ) patients.^19^ A subgroup analysis showed similar occurrence of HBsAg-loss (*P* = 0.290) and similar HBsAg dynamics between GZ and HNCH patients, also when taking treatment allocation into account (Supp. Table 3 and Supp. Figure 4A–D). HBV-DNA dynamics differed between both subgroups. In the untreated group, GZ patients had higher HBV DNA baseline levels but showed a HBV DNA-decline over the study period up to −1.40 log_10_ IU/mL (mean, SD 1.37, *P* = 0.003) after five years while the patients in the HNCH group did not (*P* = 0.159). This resulted in similar HBV-DNA levels between the groups from year 1 onwards. The opposite was found in the treated patients in whom HBV DNA levels were similar at EOT but were elevated in GZ patients compared to HCNH patients, irrespective of treatment. Of all patients attending the last follow-up visit, 11/13 (84.6%) of the untreated and 16/26 (61.5%) treated GZ patients changed to HNCH group. The reverse was seen in 3/24 (12.5%) untreated and 3/41 (7.3%) treated HCNH patients that changed to GZ at end of follow-up.

### Clinical Outcomes Long Term Follow-up

Fibrosis levels by Fibroscan measurement declined significantly over time from baseline to the end of five year follow-up in patients treated with Peg-IFN/ADV (−0.76 kPa, *P* = 0.023) and Peg-IFN/TDF (−0.43 kPa, *P* = 0.030) but not in the untreated patients (−0.32 kPa, *P* = 0.399), Supplementary Figure 5. Noteworthy, patients treated with Peg-IFN/ADV had already lower (*P* = 0.010) fibrosis levels at baseline compared to the other two groups. ALT levels declined (*P* = 0.029) from 31 U/L (median, IQR 22–48) at baseline to 27 U/L (median, IQR 21–37) at end of follow-up in the untreated patients while treated patients showed no significant decline (*P* = 0.179 and *P* = 0.227 for Peg-IFN/ADV and Peg-IFN/TDF respectively). Ten patients reached ALT levels of ≥ 5x ULN during treatment, of whom nine were treated with Peg-IFN/ADV and one with Peg-IFN/TDF. During follow-up, two of these patients achieved HBsAg-loss (20%) and two were lost to follow-up. Furthermore, one patient treated with Peg-IFN/ADV had a post treatment flare at one-year follow-up and was treated successfully with tenofovir. During Follow-up, a total of five (3.7%) patients met the criteria for NA-treatment (three from the Peg-IFN/ADV group and one in both the other groups) and none of the participants developed a HCC. One patient, with Ishak score 2 at baseline, discontinued Peg-IF/ADV treatment at week 36 due to alcohol use, switched to tenofovir monotherapy, and was diagnosed with alcoholic/drug induced cirrhosis Child-Pugh A one year after EOT.

### Adverse Events

Persistence of adverse events that developed in the initial study were monitored during follow-up. Thyroid dysregulation was initially seen in seven patients treated with Peg-IFN (5%). Of the patients without thyroid-disease before start of study (5/7), four recovered after temporary levothyroxine or tiamazole treatment. One patient was still levothyroxine-dependent after end of follow-up. Of the patients that experienced mood changes or depression under treatment, 17 of 23 completed the five-year follow-up. These psychiatric complaints had disappeared in almost all patients after EOT except for one patient (treated with Peg-IFN + ADV) who experienced persistence of mood changes and concentration problems up to the end of follow-up. Also, one patient who developed a severe depression with pseudo hallucinations at year 5 of follow-up after being without psychiatric complaints between end of Peg-IFN + ADV treatment and year 4. Causality between treatment and persistence of complaints after EOT could not be determined since this was observed in only one patient.

## Discussion

The long-term effect of combining Peg-IFN with TDF or ADV was not studied previously in a prospective randomised controlled trial. In this study, we showed that Peg-IFN + NA combination therapy for 48 weeks did not lead to a higher rate of HBsAg-loss compared to no-treatment up to 5 years after treatment, in HBeAg negative CHB patients with a low viral load.

In the recent years, several studies have reported on the effect of Peg-IFN + NA combination treatments. In contrast to our study, the vast majority of these studies included a short term follow-up^20^ and focussed on HBeAg positive patients^21^, ^22^, ^23^, ^24^, ^25^, ^26^ or patients with a high viral load.^15^^,^^27^ Often without including an untreated control group. We found two long-term follow-up studies that describe the HBsAg-loss rate after Peg-IFN and lamivudine or ADV combination therapy in patients with a high viral load.^27^^,^^28^ At five years after treatment, combination therapy had led to HBsAg-loss in respectively 12% and 17.2–19.3% of the patients. These five year HBsAg-loss rates are comparable to the percentage of HBsAg-loss found in our cohort of 18.6% (Peg-IFN/ADV) and 11.7% (Peg-IFN/TDF). This similarity was unexpected since higher HBsAg-loss rates were hypothesised in patients who have lower HBsAg levels at baseline and patients included in our study had lower HBsAg levels compared to previous studies.

Comparing outcomes by treatment allocation showed that treatment did not lead to higher rates of HBsAg-loss compared to no-treatment. This contradicted our hypothesis that the use of immune modulatory therapy could induce HBsAg-loss in patients with a low viral load. This was further ascribed by the results of Bourliere *et al.*, in which HBeAg negative patients with an undetectable viral load under continuing NA therapy did not benefit from Peg-IFN add-on therapy up to week 96.^29^ These patients were dependent on NA treatment for viral suppression rather than having an immune-mediated viral suppression such as seen in HBeAg negative low viral load patients from our cohort. It might however be argued that suppressing viral replication with NA therapy can lead to the restoration of T-cell responses,^30^ and subsequently to an immune state which is similar to that of the cohort in our study.^31^ This may explain the similarities found between the two study outcomes.

The untreated patients had a relatively high incidence of HBsAg-loss (14.7%) after follow-up. Since there were no similar trials that included an untreated control group, we compared our findings to previous natural history reports. In HBeAg negative patients with a low viral load, annual expected spontaneous HBsAg-loss rates of 0.7–2.2% have been described as well as a 5 year incidence of 4.74%.^4^^,^^11^^,^^32^ These numbers are lower compared to our findings. A possible explanation for this discrepancy might be that patients in our study had a low HBV DNA and HBsAg level at inclusion, both of which are related to a higher HBsAg-loss rates.^33^ More in detail, patients with HBV DNA levels below 2000 IU/mL have an 8.38% chance to achieve HBsAg-loss within 5 years. In our untreated cohort, the baseline HBV DNA levels were even lower with 2.76 Log10 IU/ml (approximately 575 IU/mL) which could explain the even higher HBsAg-loss rate than 8.38%. Also, characteristics such as older age, male sex, and genotype C are related to a higher incidence of HBsAg-loss but are not of much importance since our cohort was relatively young (mean 42 years) with a balanced distribution of genotype and sex.

A post-hoc analysis of our data compared patients with a strict HNCH infection and GZ patients with a higher viral load or ALT level at baseline. The majority of patients that were classified as GZ at baseline had converted in to a HNCH infection phase, both with (61.5%) and without (84.6%) treatment. This spontaneous ‘switch’ in classification by decline in viral activity has also been described by others with lower rates of 43.5–45% in respectively 5–8.3 years.^34^^,^^35^ Based on these findings, a conservative attitude towards treating GZ patients could lead to high rates of spontaneous decline in viral replicative activity and redundancy of the need for antiviral therapy.

Long-term follow-up by means of fibroscan showed a declined liver stiffness level over time in both treated arms but not in the untreated patients. This decline was limited and did not lead to an overall declined Metavir score since this was already only F0-1 at baseline. Therefore, the clinical relevance of this finding might be debatable. Especially since no increase of fibrosis was observed in both the treated and untreated patients.

After follow-up, the proportions of patients with HBsAg-loss were sufficient to examine the predictive value of baseline characteristics on the chance of HBsAg-loss within 5 years. Indeed, we found that HBsAg baseline levels and genotype A were independent predictors of HBsAg-loss. This predictive value of baseline HBsAg levels, has previously also been described in HBeAg negative patients treated with peg-IFN/NA combination therapy. Nevertheless, our finding that HBsAg-levels are predictive of HBsAg-loss irrespective of treatment (or no-treatment) allocation is new. We found a noteworthy high negative predictive value of 95%. This finding will not contribute to the selection of patients that could be treated but may offer a perspective for the long-term outcome in patients under monitoring care.

In conclusion, the long term follow up of CHB patients with low viral load, previously treated with NA/peg-IFN combination showed that the rate of HBsAg-loss overtime was similar to that of an untreated control group.

## Credit authorship contribution statement

R. Erken: Data acquisition long term follow-up, Data curation, Validation, Formal analysis, Methodology, Writing - original draft.

V.V. Loukachov: Data curation, Validation, Formal Analysis, Writing - original draft.

A. de Niet: Patient Enrollment, Conceptualization, Methodology.

L. Jansen: Patient Enrollment, Methodology, Writing - review & editing.

F. Stelma: Patient Enrollment, Data acquisition long term follow-up, Writing - review & editing.

J.T. Helder, Project administration, Data acquisition long term follow-up.

M.W. Peters, Project administration, Data acquisition long term follow-up.

H.L. Zaaijer: Resources, Supervision, Writing - review & editing.

N.A. Kootstra: Resources, Supervision, Writing - review & editing.

S.B. Willemse: Patient Enrollment, Supervision, Writing - review & editing.

H.W. Reesink: Conceptualization, Funding acquisition, Resources, Supervision, Writing - review & editing.

## Conflicts of interest

R. Erken, V.V. Loukachov, A. de Niet, L. Jansen, F. Stelma, J.T. Helder, M.W. Peters, H.L. Zaaijer and N.A. Kootstra declare no conflict of interest. S.B. Willemse has served as a speaker, a consultant or an advisory board member for AbbVie, Gilead, Thermo Fisher and has received research funding from 10.13039/100006483AbbVie, 10.13039/100005958ENYO and 10.13039/100004337Roche. H.W. Reesink has served as a consultant or an advisory board member for Springer Healthcare, ENYO, Roche, R-Pharm and Virology Education.

## Acknowledgements

We would like to express gratitude to Maarten Koot from Sanquin Diagnostics for doing virology analysis. We thank Hadassa Heidsieck for organising the patient follow-up visits. We would also like to thank Sjoerd Kuiken, Sebastiaan Weijer, Carin van Nieuwkerk, Richard Molenkamp, Bart Takkenberg, Joanne Verheij and Ulrich Beuers for their contribution to the randomised controlled trial that served as the basis for this extended long-term follow-up study.

## Funding

The initial study was funded in full by 10.13039/100004337Roche and 10.13039/501100003142Fonds NutsOhra by an unrestricted grant. The long-term follow-up study was sponsored in part by Stichting Fonds voor Bloedtransfusie-en Ander Medisch-Wetenschappelijk Onderzoek by an unrestricted grant. Funding partners had no role in study design, data collection, data analysis, data interpretation, or writing of the report.

## Appendix A Supplementary data

Consort flow diagram Consort flow diagram

Protocol Protocol
