# Supplementary material for: A Prospective Five-Year Follow-up After peg-Interferon Plus Nucleotide Analogue Treatment or no Treatment in HBeAg Negative Chronic Hepatitis B Patients
Source: J Clin Exp Hepatol. 2022 Jan 4;12(3):735–44. doi: 10.1016/j.jceh.2021.12.011 (PMC9168707; doi:10.1016/j.jceh.2021.12.011)
Supplement: Consort flow diagram [file mmc1.doc]

**Allocation**

**Analysis**

**Enrollment**

**Assigned to no treatment n= 48**

♦ Received ≥1 intervention n= 43

♦ Did not receive intervention n= 5

Did not attend any LTFU visit n=1

Did not attend any LTFU visit n=1

Did not attend any LTFU visit n=1

# Consort Flow diagram

**Follow-Up**

**At least one LTFU visit n=45**
♦ Last visit 1 year after EOT n=1
♦ Last visit 2 years after EOT n=1
♦ Last visit 3 years after EOT n=2
♦ Last visit 4 years after EOT n=4
♦ Last visit 5 years after EOT n=37

**Assigned to Peg-IFN and Adefovir n=52**

♦ Received ≥1 intervention n=46

♦ Did not receive intervention n=6

**At least one LTFU visit n=44**
♦ Last visit 1 year after EOT n=3
♦ Last visit 2 years after EOT n=3
♦ Last visit 3 years after EOT n=2
♦ Last visit 4 years after EOT n=5
♦ Last visit 5 years after EOT n=31

Randomized n = 151

**Assigned to Peg-IFN and tenofovir n=51**

♦ Received ≥1 intervention n=45

♦ Did not receive intervention n=6

**At least one LTFU visit n=42**
♦ Last visit 1 year after EOT n=0
♦ Last visit 2 years after EOT n=0
♦ Last visit 3 years after EOT n=1
♦ Last visit 4 years after EOT n=2
♦ Last visit 5 years after EOT n=39

**HBsAg positivity known at end of LTFU n=43**
♦ Attended last LTFU visit n=37
♦ Last visit >4 months and 5 years after EOT n= 4
♦ LTFU dropout after FC n= 2

**HBsAg positivity known at end of LTFU n=34**
♦ Attended last LTFU visit n=31
♦ Last visit >4 months and 5 years after EOT n=3

**HBsAg positivity known at end of
LTFU n=41**
♦ Attended last LTFU visit n=39
♦ Last visit >4 months and 5 years after EOT n=2
