# Supplementary material for: A Prospective Five-Year Follow-up After peg-Interferon Plus Nucleotide Analogue Treatment or no Treatment in HBeAg Negative Chronic Hepatitis B Patients
Source: J Clin Exp Hepatol. 2022 Jan 4;12(3):735–44. doi: 10.1016/j.jceh.2021.12.011 (PMC9168707; doi:10.1016/j.jceh.2021.12.011)
Supplement: Protocol [file mmc2.pdf]

‘A randomized prospective open-label trial for comparing combination therapy Peg-Interferon alfa-2a (PEGASYS®) and Adefovir dipivoxil (Hepsera®) and combination therapy Peg-Interferon alfa-2a (PEGASYS®) and Tenofovir disoproxil fumarate (Viread®) versus no treatment in HBeAg negative chronic hepatitis B patients with low viral load’

PROTOCOL TITLE: ' A randomized prospective open-label trial for comparing combination therapy Peg-Interferon alfa-2a (40 KD) (PEGASYS®) and Adefovir dipivoxil (Hepsera®) and combination therapy Peg-Interferon alfa-2a (PEGASYS®) and Tenofovir disoproxil fumarate (Viread®) versus no treatment in HBeAg negative chronic hepatitis B patients with low viral load'

**Short title**	**HBsAg loss in HBV patients with low HBV-DNA load, comparing ADV/PegIFN or TDF/PegIFN with no treatment. A 3-arm randomized open-label prospective trial.**
**Version**	**4**
**Date**	**16-07-2013**
**Protocol number**	**TTM16002**
**MEC nummer**	**MEC 09/139**
**Principal investigator(s)**	**Dr. H.W. Reesink**<br>**Dept. of Gastroenterology and Hepatology**<br>**Academic Medical Center, University of Amsterdam**<br>**Room G4-214 /mailbox C2-331**<br>**Meibergdreef 9**<br>**1105 AZ Amsterdam**<br>**Email: h.w.reesink@amc.nl**
**Sponsor**	**Dr. H.W. Reesink**<br>**Dept. of Gastroenterology and Hepatology**<br>**Academic Medical Center, University of Amsterdam**<br>**Room G4-214 /mailbox C2-331**<br>**Meibergdreef 9**<br>**1105 AZ Amsterdam**<br>**Email: h.w.reesink@amc.nl**

**Independent physician(s)**	**Prof. dr. U.H. Beuers**<br>**Dept. of Gastroenterology and Hepatology**<br>**Academic Medical Center, University of Amsterdam**<br>**Room G4-213**<br>**Meibergdreef 9**<br>**1105 AZ Amsterdam**<br>**Email: u.h.beuers@amc.nl**
**Laboratory sites**	**Academic Medical Center, University of Amsterdam**

PROTOCOL SIGNATURE SHEET

Name	Signature	Date
**Project leader/Principal Investigator:**<br><br>**and sponsor**	**Dr. H.W. Reesink**<br>**Dept. of Gastroenterology and Hepatology**<br>**Academic Medical Center, University of Amsterdam**<br>**Room G4-215 /mailbox C2-331**<br>**Meibergdreef 9**<br>**1105 AZ Amsterdam**<br>**Email: h.w.reesink@amc.nl**	

**Coordinating Investigator**	**Drs. F. Stelma<br>Dept. of Gastroenterology and Hepatology<br>Academic Medical Center, University of Amsterdam<br>Room G4-214 /mailbox C2-331<br>Meibergdreef 9<br>1105 AZ Amsterdam<br>Email: f.stelma@amc.nl<br>+31 20 5667805**	
**Coordinating Investigator**	**Drs. L.J. Jansen<br>Dept. of Gastroenterology and Hepatology<br>Academic Medical Center, University of Amsterdam<br>Room G4-214 /mailbox C2-331<br>Meibergdreef 9<br>1105 AZ Amsterdam<br>Email: l.jansen@amc.nl<br>+31 20 5665383**	

TABLE OF CONTENTS

1. INTRODUCTION AND RATIONALE 11
2. OBJECTIVES 14
2.1 Primary Objective: 15
2.2. Secondary Objective: 15
3 STUDY DESIGN 17
4. STUDY POPULATION 17
4.1 Population (base) 17
4.2 Inclusion criteria 17
4.3.Exclusion criteria 17
4.4 Sample size calculation 19
5. TREATMENT OF SUBJECTS 20
5.1 Investigational product/treatment 20
5.2 Use of co-intervention 21
5.3 Escape medication (if applicable) 21
6. INVESTIGATIONAL MEDICINAL PRODUCT 22
7. METHODS 34
7.1 Study parameters/endpoints 35
7.1.1. Main study parameter/endpoint 35
7.1.2. Secondary study parameters/endpoints 35
7.1.3. Other study parameters 35
7.2. Randomisation, blinding and treatment allocation 34
7.3. Study procedures 34
7.4. Withdrawal of individual subjects 34
7.4.1.Specific criteria for withdrawal 35
7.5. Replacement of individual subjects after withdrawal 35
7.6.Follow-up of subjects withdrawn from treatment 35
7.7Premature termination of the study 35
8.0 SAFETY REPORTING 36
8.1Safety Assessments 36
8.2 Section 10 WMO event 36
8.3 Adverse and serous adverse events 36
8.4 Follow-up of adverse events 39
9 STATISTICAL ANALYSIS 40
9.1 Descriptive statistics 41
9.2.Interim analysis 42
10 ETHICAL CONSIDERATIONS 43
10.1 Regulation statement 43
10.2 Recruitment and consent 43
10.3 Compensation for injury 44
10.4 Incentives 44
11. ADMINISTRATIVE ASPECTS AND PUBLICATION 44

11.1 Handling and storage of data and documents 44
11.2 Amendments..44
11.3 Annual progress report...44
11.4 End of study report...44
11.5 Public disclosure and publication policy 44
12. REFERENCES...45
13. APPENDIX 48

LIST OF ABBREVIATIONS AND RELEVANT DEFINITIONS

**ABR**	**ABR form (General Assessment and Registration form) is the application form that is required for submission to the accredited Ethics Committee (ABR = Algemene Beoordeling en Registratie)**
**AE**	**Adverse Event**
**AR**	**Adverse Reaction**
**CA**	**Competent Authority**
**CCMO**	**Central Committee on Research Involving Human Subjects**
**CV**	**Curriculum Vitae**
**DSMB**	**Data Safety Monitoring Board**
**EU**	**European Union**
**EudraCT**	**European drug regulatory affairs Clinical Trials GCP Good Clinical Practice**
**IB**	**Investigator's Brochure**
**IC**	**Informed Consent**
**IMP**	**Investigational Medicinal Product**
**IMPD**	**Investigational Medicinal Product Dossier**
**METC**	**Medical research ethics committee (MREC); in Dutch: medisch ethische toetsing commissie (METC)**
**(S)AE**	**Serious Adverse Event**
**SPC**	**Summary of Product Characteristics (in Dutch: officiële productinfomatie IB1-tekst)**
**Sponsor**	**The sponsor is the party that commissions the organisation or performance of the research, for example a pharmaceutical company, academic hospital, scientific organisation or investigator. A party that provides funding for a study but does not commission it is not regarded as the sponsor, but referred to as a subsidising party.**
**SUSAR**	**Suspected Unexpected Serious Adverse Reaction**
**Wbp**	**Personal Data Protection Act (in Dutch: Wet Bescherming Persoonsgevens)**
**WMO**	**Medical Research Involving Human Subjects Act (Wet Medisch-wetenschappelijk Onderzoek met Mensen**

SUMMARY

Rationale:

Worldwide, approximately 400 million people are chronically infected with hepatitis B virus (HBV). Chronic HBV infection increases the risk of developing cirrhosis, hepatic decompensation and hepatocellular carcinoma (HCC).(1, 2) The risk of developing hepatocellular carcinoma is highest in HBeAg positive patients with high HBV DNA levels, but still the relative risk remains 10 for HBeAg negative patients. (3) Furthermore it has been shown that when HBsAg is cleared before cirrosis has developed, the prognosis is excellent (4, 5). Recently we have shown that HBeAg negative patients with high HBV-DNA load and low baseline HBsAg levels had a significantly higher HBsAg clearance (positive predictive value of 85%) after combination therapy with peginterferon alfa2a (Peg-IFN) and adefovir. (6) Based on these results, a trial was designed to investigate whether combination of a nucleos(t)ide analogue combined with PegIFN, could also provoke a high rate of HBsAg clearance in chronic hepatitis B patients with low (HBV DNA <20,000 IU/mL) viral load.

Study design:

This is a three arm open-label prospective randomized controlled trial. 150 patients will be enrolled into the study after assessment of elegibility. All patients must have documented HBsAg positivity for longer than 6 months, HBeAg negativity, anti-HBe positivity, HBV DNA < 20,000 IU/mL and ALT < 5 * upper limit of normal. Patients with a Child Pugh class B or C will be excluded. Group 1 will consist of patients treated with Peg-IFN and adefovir, group 2 will consist of patients treated with Peg-IFN and tenofovir and group 3 will consist of untreated controls. Patients in group 1 and 2 will receive medication for the period of one year. For enrolment into the study a liverbiopsy at time of enrolment is compulsory and is advisable at end of treatment (week 48).

Study population: The study population will consist of 150 patients chronically infected with hepatitis B virus with low viral load and HBeAg negativity.

Main study parameters/endpoints:

The aim of this study is to investigate what proportion of HBeAg negative, inactive carriers of the hepatitis B virus with low (< 20,000 IU/mL) load will lose HBsAg when treated with nucleot(s)ide analogue/Peg-IFN combination therapy.

Nature and extent of the burden and risks associated with participation, benefit and group relatedness:

During a period of 1.5 year blood samples will be collected and tested at 21 defined timepoints, the number of site visits will be 21 (see appendix table 1).

Questionnaires about compliance and quality of life will be asked to be filled in. Potential physical and physiological discomfort associated with participation are the burden of undergoing a liver biopsy, taking medication, extensive site visits, extra blood samples that will be taken, adverse reactions and serious adverse reactions.

1. INTRODUCTION AND RATIONALE

Approximately 400 million individuals worldwide, 6% of the world population, are chronically infected with hepatitis B virus (HBV), and 2 billion have serological evidence of past or present HBV infection.(7)
Worldwide HBV is transmitted mostly perinatally or horizontally early during childhood, whereas in western countries infection is mainly transmitted via sexual intercourse.
Despite the availability of a safe and effective vaccine for more than two decades, HBV infection is still a major global health problem. People with chronic HBV infection are at increased risk of developing hepatic decompensation, liver cirrhosis, and hepatocellular carcinoma (HCC). It is estimated that worldwide 30% of liver cirrhosis and 25% of HCC is due to HBV infection.(8) Mortality estimation models estimated 563,000-1.000,000 deaths due to HBV infection each year, thereby making chronic hepatitis B the seventh leading cause of death in the world. (8-10) Prevalence of chronic HBV infection however is low (0.1-2.0%) in Western Europe and the USA, intermediate in Mediterranean countries and Japan (2.0-8.0%), and high in Southeast Asia and sub-Saharan Africa (8.0-20.0%) (11). The annual incidence of spontaneous HBsAg loss, signifying clearance of the virus, is only 0.1%-0.8% in patients from Southeast Asia, and 1-2% in patients from Western Europe and the USA.
Progression from acute to chronic hepatitis B is influenced by the patient’s age at acquisition of the virus. When the virus is acquired perinatally, infection becomes chronic in more than 90% of new borns. In contrast when the infection is acquired during adulthood, most acute individuals clear the infection and only approximately 5% of persons become chronically infected (12).

Figure 1 represents the natural course of chronic hepatitis B, when infection is acquired perinatally. Chronic hepatitis B can present as two distinct forms: HBeAg-positive and HBeAg-negative infection. Patients are rendered for treatment when the patient develops active disease regardless of HBeAg positivity or negativity.

In the last decade therapeutic options for chronic hepatitis B have dramatically improved, which resulted in more patients achieving a state of inactive disease. Unfortunately treatment is not yet optimal, and a cure (i.e. loss of HBsAg) is still far away.

Two types of therapies are currently available for the treatment of chronic hepatitis B:

1) nucleos(t)ide analogues, which are antiviral agents inhibiting the activity of reverse transcriptase, a viral DNA polymerase. Several nucleoside/nucleotide analogues are licensed for the treatment of HBV in the Netherlands; lamivudine, entecavir, telbivudine, which are nucleoside analogues and adefovir dipivoxil (ADF) and tenofovir disoproxil fumarate (TDF),

which are a nucleotide analogues. The mode of action of nucleotide and nucleoside analogues is the same, by inhibiting the HBV reverse transcriptase.(13-16)

2) Conventional interferon and pegylated interferon (13, 17, 18).

Interferon alpha has a dual mode of action, with both antiviral and immunomodulatory effects (19-21) and has been recommended as one of the first-line therapies for both HBeAg-positive and HBeAg-negative chronic hepatitis B patients according to the EASL guidelines 2009 (22). Pegylated interferon (Peginterferon alpha2a (Peg-IFN)) is registered for the treatment of HBeAg positive as well as HBeAg negative chronic hepatitis B. Peg-IFN can be administered once weekly due to attachment of interferon alfa-2a to 40 KD branched-chain polyethylene glycol (Peg).

Dutch guidelines advise treatment with anti-virals of all chronic hepatits B patients with HBV-DNA > $2*10^4$ IU/mL with persistent or intermittent elevation of ALT (alanine aminotransferase) above the upper limit of normal (ULN) and with moderate/severe hepatitis on biopsy using a standardized scoring system (e.g. at least grade A2 or stage F2 by METAVIR scoring), regardless if they are HBeAg positive or negative (34).

These types of therapies have suboptimal efficacy. Peg-IFN is administered once weekly subcutaneous and is associated with dose-limiting adverse events. Nucleos(t)ide analogues are better tolerated but require life long administration and therefore nucleoside analogue treatment has been associated with development of drug resistance (23).

For example, the long-term use of lamivudine promotes viral resistance at an estimated rate of 14 to 32% per year. The 5 year resistance for lamivudine is 70%, for adefovir 30%, for entecavir 1.2%, for tenofovir unknown but 0% after 2 years (24). Emergence of resistant mutants or discontinuation of antiviral therapy has been associated with acute exacerbations of liver disease with viral rebound and ALT elevations(13). Although ADF is active against lamivudine resistant mutants, long-term ADF therapy has been found to be associated with development of a, ADF-resistant, rtN236T HBV viral mutation in a small number of patients (25).

In HBeAg-positive patients, HBeAg loss/seroconversion has long been selected as the primary goal of treatment. This is because studies on the natural history of HBV indicate that, in the majority of patients with HBeAg-positive chronic hepatitis B, HBeAg loss/seroconversion (either spontaneous or induced by antiviral therapy) is closely correlated with suppression of HBV DNA, ALT normalization and disease remission (26-28).

Eradication of HBV infection is rendered difficult because stable, long enduring covalently closed circular DNA (cccDNA) and HBV DNA becomes integrated in the hepatocyte nuclei. As the course for chronic hepatitis B into developing cirrhosis/HCC is slow, the major goals of therapy are the long term prevention of progression to irreversible liver damage.

Relatively short-term follow up studies use different surrogate outcomes, which have been proven to be durable and predictive for further progression of the disease. As HBV DNA levels are associated with the progression to irreversible liver damage, the goal of treatment at present has been effective suppression of HBV replication, rather than curing the disease.

Recently however, increasing attention is paid to the loss of HBsAg as the primary goal for treatment. HBsAg loss is the most reliable efficacy parameter for both HBeAg-positive and HBeAg-negative chronic hepatitis B. Serum HBsAg is a marker of HBV infection, and antibodies against HBsAg (anti-HBs) signify recovery.

When in current strategies loss of HBsAg and anti-HBs conversion has been achieved, it is considered as the closest outcome to clinical cure of chronic hepatitis B infection. Especially in patients who clear HBsAg before they have developed cirrosis, the prognosis is excellent compared to patients who remain HBsAg positive. Treatment with nucleoside analogues for one year does not increase the loss of HBsAg for both HBeAg positive and negative patients and remains approximately 1%. One year treatment with Peg-IFN results in HBsAg loss of approximately 3% in the HBeAg positive patients and 4% in the HBeAg negative patients. (29). In a study with Peg-IFN and lamuvidine therapy for one year with patients with HBeAg positivity, in 11% of patients HBsAg surface loss was seen after 3 years of follow up.

Another long-term follow up study in HBeAg negative patients, treated with Peg-IFN with or without lamivudine for one year showed a 12% loss (both with and without lamivudine) of HBsAg 5 years post-treatment (30).

In our currently ongoing study in patients with a high viral load (HBV DNA ≥ 20,000 IU/mL) combination therapy of Peg-IFN and ADF, a rate of 23% HBsAg loss was reported after one year of therapy, which is much higher than currently is observed with other treatment regimens (6). Moreover in this study a positive predictive value of HBsAg loss of 85% was seen when baseline HBsAg levels were less than 675 IU/mL, suggesting that patients with lower baseline HBsAg levels are more susceptible to HBsAg clearance. Treatment in these patients was well tolerated, 4% developed thyroid disease which is comparable to other studies (31). Recent studies have shown that tenofovir is a more potent viral suppressor than adefovir (32). Moreover it has recently been reported that the rate of HBsAg loss in HBeAg positive patients after 2 years of tenofovir (TDF) therapy was 6% (33).

Chronic hepatitis B patients with low viral load (HBV DNA < 20,000 IU/mL) most often have inactive disease en don't require viral suppression according to Dutch guidelines (34). However in the light of the currently highly successful rate of HBsAg loss seen in our ongoing study (6) we hypothesize that a similar rate of HBsAg clearance can be achieved in HBeAg negative patients with low HBV DNA viral load, thereby focusing on cure of the disease rather than viral suppression.

In the light of the suboptimal therapies currently available e.g. the dose limiting adverse reactions of Peg-INF treatment and the development of drug resistance, it is of great practical value to establish predictors of response (or non-response) before or early in the course of anti-viral treatment to assess the success rate of antiviral agents and to guide therapeutic interventions in hepatitis B.

In this study we hypothesize that both treatment with peg-interferon and ADF or Peg-IFN and TDF in HBeAg negative chronic hepatitis B patients with low HBV DNA viral load will induce a high rate of HBsAg loss, comparable to that in patients with high viral load after treatment with ADF and Peg-IFN.

2. OBJECTIVES

2.1 Primary Objective:

The primary objective is to demonstrate the efficacy of combination therapy (Peg-IFN and adefovir or Peg-IFN and tenofovir) for inducing loss of HBsAg compared to no-treatment in HBeAg negative chronic hepatitis B patients with low viral load. HBsAg loss is defined as HBsAg level < 0.05 IU/mL.

2.2. Secondary Objective:

The secondary objectives are to evaluate:
a. the rate of HBsAg loss and anti-HBs serconversion,
b. To establish predictive markers at baseline and during the first 12 weeks of treatment for response of primary and secondary endpoints:

serologic markers
- HBsAg levels

immunologic markers
- T cell responses, cytokine production of T cells.

histologic markers
- HAI (histology activity index)
- Fibrosis grade
- HBsAg level
- cccDNA level (liver)
- HBV-DNA level (liver)

biochemical markers (livertransaminases)
- ALT
- AST
- LDH
- AF
- yGT

virologic markers
- HBsAg level
- cccDNA (plasma)
- HBV-DNA level
- HDV co-infections

Hostgenomics

DNA and RNA expression levels in PBMC's, liver

Proteomics

Possible protein candidates include: SR-B1(CD36), CD81, certain Claudins, STAT-1 and 2, ApoE and more

Inflammatory markers

CRP, neoptorin

Neoplastic markers

Rate of development of hepatocellulair carcinoma

3. STUDY DESIGN

This is a three arm open-label prospective randomized controlled trial. (n=150) The trial is designed to compare the efficacy of treatment with Peg-IFN and ADF or Peg-IFN and TDF versus no treatment for clearance of the HBsAg in chronic hepatitis B patients with low viral load.

Patients will be enrolled into the study after assessment of elegibility. Group 1 will consist of patients treated with Peg-IFN 180µg once weekly and ADF 10mg once daily (standard doses), group 2 will consist of patients treated with Peg-IFN 180µg once weekly and TDF 300mg (equivalent to 245 mg tenofovir disoproxil) once daily (standard doses) and group 3 will consist of untreated patients as negative controls.

The trial consists of a screening period of approximately 4 weeks, a 48 week treatment period, a 24 week follow-up period and a long term follow up period of 5 year. A schematic overview of the study design is presented in table 1 and 2 (see appendix).
150 patients will be randomized in a 50:50:50 ratio to one of the treatment groups. Randomization will be stratified to genotype A to optimize balance between treatment groups. All patients will receive medication for the period of 48 weeks. For enrolment into the study a liver biopsy at time of enrolment is compulsory for patients selected in the treatment groups. For enrolment of patients that are selected in the non-treatment group a liverbiopsy is optional but not compulsory for participation. In patients selected in the treatment group a second liver biopsy at week 48 is recommended for evaluation of therapeutic responses and to guide any further possible therapy. Due to the burden of undergoing a liver biopsy, the second liver biopsy is not compulsory to take part into the study. Patients that are selected in the non treatment group will not undergo a biopsy at week 48. Patients will be clinically monitored intensively according to the assessment schedule (table 1). In the long term follow up period patients will be clinically monitored each year.

4 STUDY POPULATION

4.1 Population (base)

Patients will be enrolled into the study after assessment of eligibility. Target population will consist of adult patients (≥ 18 years) with chronic hepatitis B (HBsAg positivity ≥ 6 months), HBeAg negativity,HBV DNA < 20,000 IU/ml and ALT < 5* upper limit of normal.

4.2 Inclusion criteria

1. Male and female patients ≥ 18 and ≤ 70 years of age
2. Positive HBsAg for more than 6 months.
3. Negative for HBeAg for more than 6 months.
4. HBV DNA < 20,000 IU/ml
5. Patients with chronic hepatitis B who are either naive to antiviral treatment, or have received either interferon (IFN) or nucleoside/nucleotide analogues in the past but are still positive for HBsAg.
6. Serum ALT ≤ 5 * ULN as determined by two values taken ≥14 days apart during the six months before the first dose of study drug with at least one of the determinations obtained during the screening period.
7. Negative urine or serum pregnancy test (for women of childbearing potential) documented within the 24-hour period prior to the first dose of test drug.

4.3. Exclusion criteria

1. Patients co-infected with HCV, HIV or who have decompensated liver disease, hepato-cellular carcinoma, significant cardiac disease, significant renal disease, seizure disorders or severe retinopathy.
2. Patients who have received nucleos(t)ide analogues for their chronic hepatitis B within 6 weeks before enrollment or have received Peg-IFN within 3 months before enrollment.
3. Patients must not have received any other systemic anti-viral, anti-neoplastic or immuno-modulatory treatment (including supraphysiologic doses of steroids or radiation) ≤3 months prior to the first dose of study drug or the expectation that such treatment will be needed at any time during the study.
4. Positive test at screening for anti-HAV IgM, anti-HIV, HCV RNA. (Patients that have cleared the hepatitis C virus can be included in the study)

5. Patients who are expected to need systemic antiviral therapy other than that provided by the study at any time during their participation in the study are also excluded. Exception: patients who have had a limited (≤7 day) course of acyclovir for herpetic lesions more than 1 month prior to the first administration of test drug are not excluded.
6. Evidence of decompensated liver disease (Child pugh B-C)
7. Serum total bilirubin > twice the upper limit of normal at screening
8. History or other evidence of bleeding from esophageal varices or other conditions consistent with decompensated liver disease.
9. History or other evidence of a medical condition associated with chronic liver disease other than HBV (e.g., hemochromatosis, autoimmune hepatitis, metabolic liver diseases including Wilson's disease and alfa1-antitrypsin deficiency, alcoholic liver disease, toxin exposures, thalassemia).
10. Women with ongoing pregnancy or who are breast feeding.
11. Neutrophil count <1500 cells/mm^3 or platelet count <80,000 cells/mm^3 at screening.
12. Hemoglobin < 7.1 mmol/L (< 11.5 g/dL) for females and < 7.8 mmol/L (< 12.5 g/dL) for men at screening.
13. Serum creatinine level >1.5 times the upper limit of normal at screening.
14. Unstable ongoing severe psychiatric disease, especially depression (stable patients can be included).
15. History of immunologically mediated disease (e.g., inflammatory bowel disease, idiopathic thrombocytopenic purpura, lupus erythematosus, autoimmune hemolytic anemia, scleroderma, severe psoriasis, rheumatoid arthritis).
16. History or other evidence of chronic pulmonary and cardiac disease associated with functional limitation. Severe cardiac disease (e.g., NYHA Functional Class III or IV, myocardial infarction within 6 months, ventricular tachyarrhythmias requiring ongoing treatment, unstable angina or other significant cardiovascular diseases).
17. History of a severe seizure disorder or current anticonvulsant use and clinically unstable disease.
18. Evidence of an active or suspected cancer or a history of malignancy where the risk of recurrence is ≥20% within 5 years. Patients with a lesion suspicious of hepatic malignancy on a screening imaging study will only be eligible if the likelihood of carcinoma is ≤10% following an appropriate evaluation.
19. Major organ transplantation. (patients with skin, cornea or bone transplantation are allowed to be included into the study)

20. Thyroid disease with thyroid function poorly controlled on prescribed medications. Patients with elevated thyroid stimulating hormone or T4 concentrations, with elevation of antibodies to thyroid peroxidase and any clinical manifestations of thyroid disease that are not stable on prescribed medication are excluded. Stable patients can be included.
21. History or other evidence of severe retinopathy (e.g. CMV retinitis, macula degeneration) or clinically relevant ophthalmological disorder due to diabetes mellitus or hypertension.
22. Inability or unwillingness to provide informed consent or abide by the requirements of the study.
23. History or other evidence of severe illness or any other conditions which would make the patient, in the opinion of the investigator, unsuitable for the study.
24. Patients with a value of alfa-fetoprotein >100 ng/mL are excluded, unless stability (less than 10% increase) has been documented over at least the previous 3 months.
25. Evidence of current hard drug(s) (i.e. cannabis products are allowed) and/or alcohol abuse (20g/day for women and 30g/day for men).
26. Patients included in another trial or having been given investigational drugs within 12 weeks prior to screening.

4.4 Sample size calculation

Per treatment group 44 per group (88 for both groups) is needed to achieve 81% power to detect a difference between the two treatment groups and the control group. The proportion in the Peg-IFN and adefovir group is assumed to be 0.01 under the null hypothesis and 0.20 under the alternative hypothesis. The proportion in the control group (no treatment) is 0.01. The test statistic used is the two-sided Fisher's Exact test . The significance level of the test was targeted at 0.05. A group sample of 44 persons in the control group is needed to achieve a 81% power to detect a difference between the Peg-IFN adefovir or Peg-IFN tenofovir.

Regarding a 10% drop-out due to Peg-IFN side-effects or other reasons a total of 50 patients are needed in each group.

5. TREATMENT OF SUBJECT

5.1 Investigational product/treatment

Peginterferon alfa-2a (40KD) (PEGASYS®) + Adefovir (ADF) dipivoxil (Hepsera®)
Peginterferon alfa-2a (40KD) (PEGASYS®) + Tenofovir (TDF) disoproxil fumarate (Viread®)

5.2 Use of co-intervention

All medication (prescription and over- the- counter) administered from signing of informed consent onwards until the safety follow-up visit should be recorded in the CRF.
When having heterosexual intercourse, female subjects of childbearing potential and non-vasectomized male subjects who have a female partner of childbearing potential must use a combination of 2 effective birth control methods during dosing with Peg-IFN and TDF or ADF and for 6 months after discontinuation of therapy.
Use of co-intervention (e.g. painkillers) will be to the discretion of the investigator

5.3 Escape medication

Use of escape medication (e.g. switch of antiviral therapy due to side effects) will be to the discretion of the investigator.

6. INVESTIGATIONAL MEDICINAL PRODUCT

6.1. Name and description of investigational medicinal product

6.2. Product Information

Peginterferon alfa-2a (40KD) (PEGASYS®)

Summary of findings from non-clinical and clinical studies

Peginterferon alfa-2a (40KD) (PEGASYS®) was developed through the process of pegylation, whereby a large, 40-kDa, branched-chain polyethylene glycol (PEG) molecule is attached to the base interferon alfa-2a molecule to produce a drug with a prolonged half-life. The size of the PEG molecule and its structure are factors affecting the pharmacokinetic properties of pegylated interferons. The attachment of a 40-KD, branched PEG moiety to interferon alfa-2a results in a drug with a once weekly frequency of administration which maintains effective concentrations of peginterferon alfa-2a (40KD) throughout the dosing interval while substantially reducing the peak-to-trough ratio. Hence, sharp increases to peak serum concentrations can be avoided, which are linked to a number of adverse events associated with thrice-weekly IFNα dosing.(35)

PEGASYS®, as monotherapy and in combination with ribavirin has been approved for use in patients with chronic hepatitis C (36).

PEGASYS® was recently approved in the EU for treatment of HBeAg positive as well as HBeAg-negative chronic hepatitis B. The clinical development of PEGASYS® for chronic hepatitis B consists of a phase II study in patients with HBeAg-positive HBV (37) and two pivotal phase III trials in HBeAg-positive and HBeAg-negative disease (38). The HBeAg-positive study results were presented in 2004.

The primary aim of the phase II study was to compare the efficacy and safety of PEGASYS® administered once weekly for 24 weeks at 90 µg, 180 µg and 270 µg with conventional IFNα-2a 4.5 MIU thrice weekly in patients with HBeAg-positive chronic hepatitis B.

A total of 194 adult patients were enrolled in this randomized, open-label, multicenter study. Included patients were IFNα naïve and had received less than 6 months treatment with nucleoside/nucleotide analogues and/or had not received nucleoside/nucleotide therapy in the 6 months prior to the study. Patients with decompensated cirrhosis were excluded. All patients were serum positive for HBeAg, HBsAg and had viral loads >500,000 HBV DNA cop/mL. Chronic hepatitis B status was confirmed by liver biopsy and, at the time of screening, all patients had baseline ALAT levels over 2-fold greater but <10 x ULN. Patients

were randomized to four treatments arms and treated for 24 weeks with either conventional IFNα-2a or with one of three doses of PEGASYS®. All patients were monitored for an additional treatment-free 24-week follow-up period.

The percentages of patients achieving a combined response (HBeAg loss, HBV DNA <500,000 cop/mL, ALAT normalization) at end-of-follow-up (week 48) were 12%, 27%, 28% and 19% for conventional IFNα-2a, PEGASYS® 90 μg, 180 μg and 270 μg once weekly, respectively. When the data from all PEGASYS® doses were pooled, the combined response rate was 24% and this was statistically significantly different compared with conventional IFNα-2a (P=0.036). In terms of on-treatment HBV DNA suppression, a PEGASYS® dose of 180 μg gave a greater response at the end of week 24. At week 24, HBV DNA levels had not yet reached a plateau but continued to decline (37). Of the patients treated with 180 μg PEGASYS® 33% had HBeAg seroconversion and 35% had HBeAg loss after 24 weeks of treatment and 24 weeks follow-up.

Advantages of PEGASYS® over interferon alfa-2a were noted also in the subgroups of patients with difficult-to-treat disease such as those with liver cirrhosis, low ALT, and/or high HBV DNA at baseline as well as patients with HBV genotype C (37).

Overall, the data from this phase II study demonstrates that PEGASYS® has substantially higher efficacy than conventional IFNα-2a in patients with HBeAg-positive CHB .

Two phase III, randomized clinical trials with PEGASYS® in chronic hepatitis B have been performed. These trials investigated the potential of PEGASYS® (180 mcg once weekly) alone and in combination with lamivudine (100 mg daily) in the treatment of HBeAg-positive and HBeAg-negative CHB. A treatment duration of 48 weeks was proposed for the phase III trials as a result of the phase II data, which demonstrated a continuing decline in HBV DNA levels at week 24.

The principal objective of the first phase III study was to compare the efficacy and safety of PEGASYS® 180 µg once weekly with and without lamivudine 100 mg daily, to that of lamivudine 100 mg daily in the treatment of HBeAg-positive patients with CHB. A total of 814 adult patients have been enrolled and they have been randomized to one of the three treatment arms receiving 48 weeks of anti-viral therapy and monitored for a further 24 weeks post-treatment. At end of follow-up HbeAg seroconversion and HBV-DNA < 100,000 cop/mL was observed in 32 % of patients treated with Pegasys® with placebo (p<0.001), 27 % (p<0.023) with Pegasys® and Lamivudine and 19 % with Lamivudine alone. Also HbeAg loss and ALT normalization was significantly higher in 2 groups treated with Pegasys® than in the group with Lamivudine alone (38).

The aim of the second phase III study was to compare the efficacy and safety of PEGASYS® 180 µg once weekly with and without lamivudine 100 mg daily, to that of lamivudine 100 mg daily in the treatment of HBeAg-negative patients with CHB. The 537 adult patients in this trial have been randomized to one of the three treatment arms. All patients have received 48 weeks of anti-viral treatment and have been monitored for a further 24 weeks post-treatment period.

After 24 weeks of follow-up, the percentage of patents with normalization of alanine aminotransferase levels or hepatitis B virus (HBV) DNA levels below 20,000 IU/mL was significantly higher with peginterferon alfa-2a monotherapy (59 % and 43 % respectively) and peginterferon alfa-2a plus lamivudine (60 % and 44 %) than with lamivudine monotherapy (44 %, P=0.004 and P=0.003 respectively; and 29 %, P=0.007 and P=0.003 respectively). At the end of follow-up rates of sustained suppression of HBV DNA to below 400 copies per milliliter were 19 % with peginterferon alfa-2a monotherapy, 20 % with combination therapy and 7 % with lamivudine alone (P<0.001 for both comparisons with lamivudine alone). Loss of hepatitis B surface antigen occurred in 12 patients in the peginterferon groups, as compared with 0 patients in the group given lamivudine alone.

At end of follow-up (week 72), using the intent to treat analysis, the percentage with normalised ALAT or HBV-DNA (< 20,000 cop/mL) was significantly higher in the PEGASYS® plus placebo and PEGASYS® plus lamivudine groups than in the lamivudine group (38) .

In multivariate analyses, high ALT, low HBV-DNA and low HBeAg levels at baseline were significant predictors of HBeAg seroconversion (P< 0.001).In HBeAg-positive CHB, marked ALT flares during INF-based treatment have been associated with HBeAg seroconversion and viral suppression. Marked on-treatment ALT elevations were generally more frequent with peginterferon alfa-2a monotherapy and were more common in patients with HBeAg seroconversion 24 weeks after the end of treatment (37, 39).

<u>Summary of known and potential risks and benefits</u>

PEGASYS can cause a broad variety of serious adverse reactions . The most common life-threatening or fatal events induced or aggravated by PEGASYS were depression, suicide, relapse of drug abuse/overdose, and bacterial infections, each occurring at a frequency of < 1%. Hepatic decompensation occurred in 2% (10/574) of CHC/HIV patients .

In clinical trials of 48 week treatment duration, the adverse event profile of PEGASYS® in chronic hepatitis B was similar to that seen in chronic hepatitis C PEGASYS® monotherapy

use, except for exacerbations of hepatitis. Six percent of PEGASYS® treated patients in the hepatitis B studies experienced one or more serious adverse events.

The most common or important serious adverse events in the hepatitis B studies were infections (sepsis, appendicitis, tuberculosis, influenza), hepatitis B flares, anaphylactic shock, thrombotic thrombocytopenic purpura.

The most commonly observed adverse reactions were pyrexia (54% vs. 4%), headache (27% vs. 9%), fatigue (24% vs. 10%), myalgia (26% vs. 4%), alopecia (18% vs. 2%), and anorexia (16% vs. 3%) in the PEGASYS and lamivudine groups respectively.

Overall 5% of hepatitis B patients discontinued PEGASYS therapy and 40% of patients required modification of PEGASYS dose. The most common reason for dose modification in patients receiving PEGASYS therapy was for laboratory abnormalities including neutropenia (20%), thrombocytopenia (13%), and ALT disorders (11%).

Description and justification of route of administration and dosage

The recommended dose of PEGASYS monotherapy for hepatitis B is 180 µg (1.0 Ml vial or 0.5 mL prefilled syringe) once weekly for 48 weeks by subcutaneous administration in the abdomen or thigh.

When dose modification is required for moderate to severe adverse reactions (clinical and/or laboratory), initial dose reduction to 135 µg (which is 0.75 mL for the vials or adjustment to the corresponding graduation mark for the syringes) is generally adequate. However, in some cases, dose reduction to 90 µg (which is 0.5 mL for the vials or adjustment to the corresponding graduation mark for the syringes) may be needed. Following improvement of the adverse reaction, re-escalation of the dose may be considered

Dosages, dosage modifications and method of administration

Hematological:

Dose reduction from 180µg to 135µg is required if absolute neutrophil counts are < 750/mm^3. If ANC < 500/mm^3, treatment should be suspended until ANC values return to more than 1000/mm^3. Treatment must be reinstituted at 90 µg and monitor ANC needs to be monitored. When platelet counts are < 50,000/mm^3, dosage must be reduced to 90 µg. Treatment must be discontinued if platelet count < 25,000/mm^3.

Psychiatrical

Dose reduction from 180µg to 135µg is required in cases of moderate depression. Treatment should be discontinued in severe depression.

Renal Function

In patients with end-stage renal disease requiring hemodialysis, dose reduction to 135 µg PEGASYS is recommended. Signs and symptoms of interferon toxicity should be closely monitored.

Liver Function

If ALT increases are progressive despite dose reduction or accompanied by increased bilirubin or evidence of hepatic decompensation, therapy should be immediately discontinued.

In chronic hepatitis C patients with progressive ALT increases above baseline values, the dose of PEGASYS should be reduced to 135 µg and more frequent monitoring of liver function should be performed. After PEGASYS dose reduction or withholding, therapy can be resumed after ALT flares subside.

In chronic hepatitis B patients with elevations in ALT (> 5 x ULN), more frequent monitoring of liver function should be performed and consideration should be given to either reducing the dose of PEGASYS to 135 µg or temporarily discontinuing treatment. After PEGASYS dose reduction or withholding, therapy can be resumed after ALT flares subside.

In patients with persistent, severe (ALT > 10 times above the upper limit of normal) hepatitis B flares, consideration should be given to discontinuation of treatment. (35).

Adefovir (ADF) dipivoxil (Hepsera®)

Summary of findings from non-clinical and clinical studies

Adefovir (ADF) dipivoxil (Hepsera®) is a synthetic adenine nucleotide analogue that has recently been licensed for the treatment of CHB in the United States and Europe.

In vitro, the concentration of adefovir required to inhibit 50% of HBV DNA synthesis ranged from 0.2-2.5 µmol/L in a variety of HBV DNA-producing human hepatoma cell lines. Additive antiviral effects were observed when adefovir was combined with lamivudine, or with one of the experimental drugs entecavir or telbivudine.

Large, pacebo-controlled clinical trials have shown potent activity against HBV in both HBeAg-positive and HBeAg-negative patients that is associated with significant biochemical, virologic, and histologic improvement. Moreover, resistance to this agent has not been observed in up to 48 weeks of therapy. The drug has been shown to be effective both in wild-type and lamivudine-resistant forms of HBV both in vitro and in vivo. Clinical trials to date

show that virologic and biochemical improvement are observed after adefovir dipivoxil is added to ongoing lamivudine therapy in lamivudine-resistant patients or when adefovir dipivoxil is administered as monotherapy (15, 16, 40). In HBeAg positive chronic hepatits B patients (n=515) ADF given in dosages of 10 mg, 30 mg or placebo per day for 48 weeks showed that after 48 weeks of treatment, significantly more patients who received 10 mg or 30 mg of ADF dipivoxil per day than who received placebo had histologic improvement (53 percent [P<0.001], 59 percent [P<0.001], and 25 percent, respectively), a reduction in serum HBV DNA levels (by a median of 3.52 [P<0.001], 4.76 [P<0.001]. and 0.55 log cop/mL, respectively), undetectable levels (fewer than 400 cop/mL) of serum HBV DNA (21 percent [P<0.001], 39 percent [P<0.001], and 0 percent, respectively), normalization of ALAT levels (48 percent [P<0.001], 55 percent [P<0.001], and 16 percent, respectively), and HBeAg seroconversion (12 percent [P=0.0491], 14 percent [P=0.01], and 6 percent, respectively). No ADF-associated resistance mutations were identified in the HBV DNA polymerase gene. The safety profile of the 10-mg dose of ADF dipivoxil was similar to that of placebo; however, there was a higher frequency of adverse events and renal laboratory abnormalities in the group given 30 mg of ADF dipivoxil per day (15).

In HBeAg negative CHB patients (n=185) ADF given in a dosage of 10 mg or placebo once daily for 48 weeks shows that at week 48, 64 percent of patients who had base-line liver-biopsy specimens available in the ADF dipivoxil group had improvement in histologic liver abnormalities (77 of 121), as compared with 33 percent of patients in the placebo group (19 of 57, p<0.001). Serum hepatitis B virus (HBV) DNA levels were reduced to fewer than 400 cop/mL in 51 percent of patients in the ADF dipivoxil group (63 of 123) and in 0 percent of those in the placebo group 0 of 61, p<0.001). The median decrease in log-transformed HBV DNA levels was greater with ADF dipivoxil treatment than with placebo (3.91 vs. 1.35 log cop/mL, P<0.001). ALAT levels had normalized at week 48 in 72 percent of patients receiving ADF dipivoxil (84 of 116), as compared with 29 percent of those receiving placebo (17 of 59, P<0.001). No HBV polymerase mutations associated with resistance to ADF were identified. The safety profile of ADF dipivoxil was similar to that of placebo (16).

Follow-up results after 48 weeks of treatment with 10 mg AFD were presented at the ISVHLD 2003 meeting in Sydney [45]. At the end of 24 weeks follow-up without treatment, 5 % of the ADF group had undetectable HBV-DNA levels (HBV-DNA < 1000 cop/mL). After 48 weeks of follow-up 8% had undetectable HBV-DNA levels (HBV-DNA < 1000 cop/mL).

Two studies (40, 41) showed that in chronic hepatitis B with compensated and decompensated liver disease due to YMDD related Lamivudine resistance, ADF 10 mg once daily given for 48-52 weeks resulted in a significant virological and biochemical improvement.

Summary of known and potential risks and benefits

Adverse reactions to HEPSERA identified from placebo-controlled and open label studies include the following: asthenia, headache, abdominal pain, diarrhea, nausea, dyspepsia, flatulence, increased creatinine, and hypophosphatemia.

Adefovir dipivoxil 10 mg/day is generally well tolerated in patients with chronic HBV infection; there were no marked increases in adverse events or laboratory abnormalities compared with placebo in a pooled analysis of 48-week data from two double-blind trials. Asthenia and diarrhoea occurred more frequently than with placebo in one of the placebo-controlled studies, while headache and abdominal pain occurred more frequently in the other study. Within 48 weeks 2% of patients from one study and no patients in the other study discontinued treatment because of adverse events.

Nephrotoxicity characterized by a delayed onset of gradual increases in serum creatinine and decreases in serum phosphorus was historically shown to be the treatment-limiting toxicity of adefovir dipivoxil therapy at substantially higher doses in HIV-infected patients (60 and 120 mg daily) and in chronic hepatitis B patients (30 mg daily). Chronic administration of HEPSERA (10 mg once daily) may result in delayed nephrotoxicity. The overall risk of nephrotoxicity in patients with adequate renal function is low. However, this is of special importance in patients at risk of or having underlying renal dysfunction and patients taking concomitant nephrotoxic agents such as cyclosporine, tacrolimus, aminoglycosides, vancomycin and non-steroidal anti-inflammatory drugs It is important to monitor renal function for all patients during treatment with HEPSERA, particularly for those with pre-existing or other risks for renal impairment. Patients with renal insufficiency at baseline or during treatment may require dose adjustment. The risks and benefits of HEPSERA treatment should be carefully evaluated prior to discontinuing HEPSERA in a patient with treatment-emergent nephrotoxicity.

Severe acute exacerbation of hepatitis has been reported in patients who have discontinued anti-hepatitis B therapy, including therapy with HEPSERA. Hepatic function should be monitored at repeated intervals with both clinical and laboratory follow-up for at least several months in patients who discontinue HEPSERA. If appropriate, resumption of anti-hepatitis B therapy may be warranted.

In clinical trials of HEPSERA, exacerbations of hepatitis (ALT elevations 10 times the upper limit of normal or greater) occurred in up to 25% of patients after discontinuation of

HEPSERA. These events were identified in studies GS-98-437 and GS-98-438 (N=492). Most of these events occurred within 12 weeks of drug discontinuation. These exacerbations generally occurred in the absence of HBeAg seroconversion, and presented as serum ALT elevations in addition to re-emergence of viral replication. In the HBeAg-positive and HBeAg-negative studies in patients with compensated liver function, the exacerbations were not generally accompanied by hepatic decompensation. However, patients with advanced liver disease or cirrhosis may be at higher risk for hepatic decompensation. Although most events appear to have been self-limited or resolved with re-initiation of treatment, severe hepatitis exacerbations, including fatalities, have been reported. Therefore, patients should be closely monitored after stopping treatment.

Prior to initiating HEPSERA therapy, HIV antibody testing should be offered to all patients. Treatment with anti-hepatitis B therapies, such as HEPSERA, that have activity against HIV in a chronic hepatitis B patient with unrecognized or untreated HIV infection may result in emergence of HIV resistance. HEPSERA has not been shown to suppress HIV RNA in patients; however, there are limited data on the use of HEPSERA to treat patients with chronic hepatitis B co-infected with HIV.

Lactic acidosis and severe hepatomegaly with steatosis, including fatal cases, have been reported with the use of nucleoside analogs alone or in combination with antiretrovirals.

A majority of these cases have been in women. Obesity and prolonged nucleoside exposure may be risk factors. Particular caution should be exercised when administering nucleoside analogs to any patient with known risk factors for liver disease; however, cases have also been reported in patients with no known risk factors. Treatment with HEPSERA should be suspended in any patient who develops clinical or laboratory findings suggestive of lactic acidosis or pronounced hepatotoxicity (which may include hepatomegaly and steatosis even in the absence of marked transaminase elevations).

Description and justification of route of administration, dosages, dosage modifications and method of administration

The recommended dose of HEPSERA in chronic hepatitis B patients for patients ≥12 years of age with adequate renal function is 10 mg, once daily, taken orally, without regard to food. The optimal duration of treatment is unknown (42).

Adefovir dipivoxil is absorbed rapidly following oral administration. The drug is cleaved to adefovir which, in turn, is phosphorylated intracellularly to adefovir diphosphate (the active moiety). Following a single dose of adefovir dipivoxil, the bioavailability of adefovir was 59%.

Adefovir is excreted renally as unchanged drug (steady-state renal clearance [CL_R] 154 mL/h/kg). The median steady-state terminal elimination half-life was ≅7 hours in 14 patients with chronic hepatitis B. In patients with moderate to severe renal dysfunction, the systemic exposure of adefovir increased and the CL_R decreased.

In healthy volunteers, there were no clinically relevant drug interactions when lamivudine, paracetamol (acetaminophen), ibuprofen or cotrimoxazole (trimethoprim/sulfamethoxazole) were coadministered with adefovir dipivoxil.

Significantly increased drug exposures were seen when HEPSERA was administered to adult patients with renal impairment. Therefore, the dosing interval of HEPSERA should be adjusted in adult patients with baseline creatinine clearance <50 mL/min using the following suggested guidelines (see Table 4). The safety and effectiveness of these dosing interval adjustment guidelines have not been clinically evaluated. Renal function should be monitored in all patients; patients receiving concomitant drugs that are excreted renally or known to affect renal function should be closely monitored (43). Additionally, it is important to note that these guidelines were derived from data in patients with pre-existing renal impairment at baseline. They may not be appropriate for patients in whom renal insufficiency evolves during treatment with HEPSERA. Therefore, clinical response to treatment and renal function should be closely monitored in these patients. (42). Patients who discontinue adefovir dipivoxil should be closely monitored for exacerbation of hepatitis (43).

	**Creatinine Clearance (mL/min)Creatinine clearance calculated by Cockcroft-Gault method using lean or ideal body weight.**			
	**≥50**	**30–49**	**10–29**	**Hemodialysis Patients**
Recommended dose and dosing interval	10 mg every 24 hours	10 mg every 48 hours	10 mg every 72 hours	10 mg every 7 days following dialysis

Table 4.

The pharmacokinetics of adefovir have not been evaluated in non-hemodialysis patients with creatinine clearance <10 mL/min; therefore, no dosing recommendation is available for these patients.

No clinical data are available to make dosing recommendations in adolescent patients with renal insufficiency

Tenofovir disoproxil fumarate (Viread®)

Summary of findings from non-clinical and clinical studies

Tenofovir disoproxil fumarate (VIREAD®), the oral prodrug of tenofovir, is a nucleotide analog reverse transcriptase inhibitor with activity against HIV and HBV. (G. alvarez-uria, british hiv association 2009) For this reason it has been widely used in HIV-infected patients with HBV coinfection.

The pharmacological action is exerted through inhibiting viral polymerase- reverse transcriptase by direct binding, and after incorporation into DNA, by termination of the DNA chain due to the absence of a requisite 3’hydroxyl on the tenofovir molecule.

Functioning of the enzyme polymerase (reverse transcriptase) is necessary for the replication of HIV and HBV by producing viral DNA. It remains active against lamivudine-resistant HBV, and it has known activity against HBV both in patients with HBV monoinfection and in patients with HIV-1 and HBV coinfection.

Tenofovir is structurally similar to adefovir. In vitro studies showed that tenofovir and adefovir are equipotent. Because tenofovir appears to be less nephrotoxic, the approved dose is much higher than that of adefovir, 300mg vs 10mg daily. This may explain why tenofovir has more potent antiviral activity in clinical studies. Tenofovir is generally well tolerated but it has been rarely reported to cause Fanconi syndrome and renal insufficiency (44).

Tenofovir DF, is currently approved in the United States and more than 50 other countries for the treatment of humon-immunodeficiency virus type 1 (HIV-1), and it was recently approved for the treatment of chronic HBV infection in the United States, Canada, Europe, Australia and Turkey.

Recently two fase 3 studies were performed to compare the safety and efficacy of tenofovir DF with adefovir dipivoxil in HBeAg-negative or HBeAg positive patients (32). In these studies among patients with chronic HBV infection, tenofovir DF at a daily dose of 300mg

had superior antiviral efficacy with a similar safety profile as compared with adefovir dipivoxil at a daily dose of 10mg through week 48. The primairy endpoint of both HBV DNA level of less than 400 copies/ml and histologic improvement was reached in 71% vs. 49% among HBeAg negative patients and 67% vs. 12% among HBeAg positive patients.
Another recent long-term follow-up study of 34 months showed that tenofovir DF was able to controle HBV replication in most HIV-coinfected patients, regardless of previous lamivudine treatment. A sustained virologic respons rate of HBV DNA was achieved in 83%.

Summary of known and potential risks and benefits

Lactic acidosis and severe hepatomegaly with steatosis, including fatal cases, have been reported with the use of nucleoside analogs, including VIREAD, in combination with other antiretrovirals. A majority of these cases have been in women. Obesity and prolonged nucleoside exposure may be risk factors. Particular caution should be exercised when administering nucleoside analogs to any patient with known risk factors for liver disease; however, cases have also been reported in patients with no known risk factors. Treatment with VIREAD should be suspended in any patient who develops clinical or laboratory findings suggestive of lactic acidosis or pronounced hepatotoxicity (which may include hepatomegaly and steatosis even in the absence of marked transaminase elevations).

Discontinuation of anti-HBV therapy, including VIREAD, may be associated with severe acute exacerbations of hepatitis. Patients infected with HBV who discontinue VIREAD should be closely monitored with both clinical and laboratory follow-up for at least several months after stopping treatment. If appropriate, resumption of anti-hepatitis B therapy may be warranted.

Tenofovir is principally eliminated by the kidney. Renal impairment, including cases of acute renal failure and Fanconi syndrome (renal tubular injury with severe hypophosphatemia), has been reported with the use of VIREAD .
It is recommended that creatinine clearance be calculated in all patients prior to initiating therapy and as clinically appropriate during therapy with VIREAD. Routine monitoring of calculated creatinine clearance and serum phosphorus should be performed in patients at risk for renal impairment.
Dosing interval adjustment of VIREAD and close monitoring of renal function are recommended in all patients with creatinine clearance <50 mL/min.
No safety or efficacy data are available in patients with renal

impairment who received VIREAD using these dosing guidelines, so the potential benefit of VIREAD therapy should be assessed against the potential risk of renal toxicity. VIREAD should be avoided with concurrent or recent use of a nephrotoxic agent.

Description and justification of route of administration and dosage

Dosages, dosage modifications and method of administration
For the treatment of HIV-1 or chronic hepatitis B: The dose of VIREAD is 300 mg once daily taken orally, without regard to food.
In the treatment of chronic hepatitis B, the optimal duration of treatment is unknown.

Significantly increased drug exposures occurred when VIREAD was administered to patients with moderate to severe renal impairment.
Therefore, the dosing interval of VIREAD should be adjusted in patients with baseline creatinine clearance <50 mL/min. Dosing interval needs to be adjusted to every 48 hours or every 72 to 96 hours in patients with a creatinine clearance of 30-49 or 10-29 ml/min respectively. In hemodialysis patients dose should be given every 7 days or after a total of approximately 12 hours of dialysis. (Generally once weekly assuming three hemodialysis sessions a week of approximately 4 hours duration).
These dosing interval recommendations are based on modeling of single-dose pharmacokinetic data in non-HIV and non-HBV infected subjects with varying degrees of renal impairment, including end-stage renal disease requiring hemodialysis. The safety and effectiveness of these dosing interval adjustment recommendations have not been clinically evaluated in patients with moderate or severe renal impairment, therefore clinical response to treatment and renal function should be closely monitored in these patients
No dose adjustment is necessary for patients with mild renal impairment (creatinine clearance 50–80 mL/min). Routine monitoring of calculated creatinine clearance and serum phosphorus should be performed in patients with mild renal impairment

The pharmacokinetics of tenofovir have not been evaluated in non-hemodialysis patients with creatinine clearance <10 mL/min; therefore, no dosing recommendation is available for these patients.

VIREAD is available as tablets. Each tablet contains 300 mg of tenofovir disoproxil fumarate, which is equivalent to 245 mg of tenofovir disoproxil. The tablets are almondshaped, light blue, film-coated, and debossed with “GILEAD” and “4331” on one side

and with “300” on the other side (45).

No preparation and labelling of Investigational Medicinal Products is applicable to all of the investigational products.

Drug accountability

The investigator or the hospital pharmacist must maintain an adequate record of the receipt of all trial supplies. Dispensation and return, or if applicable, destruction, of the study medication must be documented by using the appropriate forms. All these records must be available for inspection at any time.

During the trial compliance will be assessed by counting returned dosage units. Subject will also be asked to record their drug intake in a diary.

7. METHODS

7.1 Study parameters/endpoints

7.1.1. Main study parameter/endpoint

The primary objective is to demonstrate the superior efficacy of combination therapy (Peg-IFN and adefovir or Peg-IFN and tenofovir) in loss of HBsAg compared to no treatment in subjects with chronic HBV and low viral loads.

HBsAg loss is defined as HBsAg level < 0.05 IU/mL.

7.1.2. Secondary study parameters/endpoints

The secondary objectives are to evaluate:

a. the rate of HBsAg loss and anti-HBs serconversion,

b. To establish predictive markers for response at baseline and during the first 12 weeks of treatment. (see paragraph 2. objectives)

7.1.3. Other study parameters

Baseline characteristics will be documented of all patients. Age, weight, sex, medical history, cause of infection, interferon naïve, previous treatment, histology activity index, ISHAK fibrosis score, prescence of cirrhosis, ethnicity will be documented.

7.2. Randomisation, blinding and treatment allocation

Patients will be randomized unblinded to one of the 3 groups. A stratification for hepatitis B genotype A will be performed.

7.3. Study procedures

Study procedures are listed in table 2.

7.4. Withdrawal of individual subjects

Subjects can leave the study at any time for any reason if they wish to do so without any consequences. The investigator can decide to withdraw a subject from the study for urgent medical reasons.

7.4.1.Specific criteria for withdrawal

Subjects may be withdrawn from the treatment if:

1. a serious side effect occurs.

2. they fail to comply with the protocol requirements or to cooperate with the investigator.

Subjects must discontinue all study medication or have their treatment modified for the following reasons:

1. all study medication must be discontinued and a subject must be withdrawn from the trial if he/she withdraws informed consent.
2. all study medication must be discontinued if a subject has a positive pregnancy test,or if the subject/partner is non-compliant with contraception requirements.

7.5. Replacement of individual subjects after withdrawal

After withdrawal of an individual subjects no replacement will take place, 10% drop out is calculated in the power of this study.

7.6.Follow-up of subjects withdrawn from treatment

Follow up of subject withdrawn from treatment will be done according the EASL HBV guidelines.

7.7.Premature termination of the study

The study will be terminated prematurely in case of serious adverse events.

8.0 SAFETY REPORTING

8.1 Safety Assessments

Safety assessments will be performed during screening, at baseline, at weeks 1, 2, 4, 6, 8, 12 and then every 6 weeks throughout the 48 weeks treatment period. Safety assessments will continue at weeks 50, 52, 56, 60 and 72 during the follow up period, as outlined in Section 4.1. Patients prematurely discontinued from test drug therapy will have a safety assessment 4 and 12 weeks after their last dose of study medication. Clinically significant laboratory abnormalities should prompt repeat measures no less frequently than every 4 weeks or in shorter intervals as clinically indicated, with appropriate clinical management, until values return to normal or baseline levels.

Measures of safety will include assessment of AEs, vital signs (systolic and diastolic blood pressure and pulse rate), and laboratory tests in addition to those carried out for efficacy as well as documentation of dose adjustments and premature withdrawals from treatment for safety or tolerability reasons. Patients with preexisting ophthalmologic disorders (e.g. diabetic or hypertensive retinopathy) should receive periodic ophthalmologic exams during therapy.

8.2 Section 10 WMO event

In accordance to section 10, subsection 1, of the WMO, the investigator will inform the subjects and the reviewing accredited METC if anything occurs, on the basis of which it appears that the disadvantages of participation may be significantly greater than was foreseen in the research proposal. The study will be suspended pending further review by the accredited METC, except insofar as suspension would jeopardise the subjects' health. The investigator will take care that all subjects are kept informed.

8.3. Adverse and serious adverse events

Adverse events are defined as any undesirable experience occurring to a subject during a clinical trial, whether or not considered related to the investigational drug.

A serious adverse event is any untoward medical occurrence or effect that at any dose results in death;

- is life threatening (at the time of the event);
- requires hospitalisation or prolongation of existing inpatients' hospitalisation;
- results in persistent or significant disability or incapacity;
- is a congenital anomaly or birth defect;

- is a new event of the trial likely to affect the safety of the subjects, such as an unexpected outcome of an adverse reaction, lack of efficacy of an IMP used for the treatment of a life threatening disease, major safety finding from a newly completed animal study, etc.

Medical and scientific judgment should be exercised in deciding whether expedited reporting is appropriate in other situations, such as important medical events that may not be immediately life-threatening or result in death or hospitalization but may jeopardize the patient or may require intervention to prevent one of the outcomes listed in the definitions above. These situations should also usually be considered serious.

The term severe is a measure of intensity, thus a severe adverse event is not necessarily serious. For example, nausea of several hours' duration may be rated as severe, but may not be clinically serious.

A death occurring during the study or which comes to the attention of the investigator within 4 weeks after stopping the treatment or during the 24 weeks of protocol-defined follow-up period, whether considered treatment-related or not, must be reported.

Any pregnancy which occurs during a clinical study with an investigational drug must be reported as an SAE for tracking purposes. All pregnancies which are identified during this study need to be followed to conclusion and outcome reported.

Female patients should immediately inform the Investigator of any pregnancies and should be instructed by the Investigator to stop taking study medication. (Pregnancies occurring up to 3 months after the completion of the study must also be reported to the Investigator). The Investigator should counsel the patient, discuss the risks of continuing with the pregnancy and the possible effects on the fetus. Monitoring of the patient should continue until the conclusion of the pregnancy.

Such preliminary reports will be followed by detailed descriptions later which will include copies of hospital case reports, autopsy reports and other documents when requested and applicable.

For serious and all other AEs, the following must be assessed and recorded on the AE page of the CRF: intensity, relationship to test substance, action taken regarding test substance, and outcome to date.

On the SAE form a drug/event relationship must be provided for each study medication (Yes or No) and each event. "Yes" must be entered for a causality of remote, possible, probable,

i.e., there is a reasonable suspected causal relationship, or causality is unknown. No is to be entered for a causality of not related.

The investigator will notify the Ethics Committee (METC) of such an event in writing in accordance with local laws and regulations. The investigator will notify the Health Authorities. The definitions for and procedures for reporting SAEs to Health Authorities will be taken from the ICH-GCP and the Dutch law 'Wet medisch-wetenschappelijk onderzoek met mensen' (WMO).

8.3.1. uspected unexpected serious adverse reactions (SUSAR)

Adverse reactions are all untoward and unintended responses to an investigational product related to any dose administered.

Unexpected adverse reactions are adverse reactions, of which the nature, or severity, is not consistent with the applicable product information (e.g. Investigator's Brochure for an unapproved IMP or Summary of Product Characteristics (SPC) for an authorised medicinal product).

The investigator will report expedited the following SUSARs to the METC:

- SUSARs that have arisen in the clinical trial that was assessed by the METC;
- SUSARs that have arisen in other clinical trial of the same sponsor and with the same medicinal product, and that could have consequences for the safety of the subjects involved in the clinical trial that was assessed by the METC.

The remaining SUSARs are recorded in an overview list (line-listing) that will be submitted once every half year to the METC. This line-listing provides an overview of all SUSARs from the study medicine, accompanied by a brief report highlighting the main points of concern. The sponsor will report expedited all SUSARs to the competent authority, the Medicine Evaluation Board and the competent authorities in other Member States.

The expedited reporting will occur not later than 15 days after the investigator has first knowledge of the adverse reactions. For fatal or life threatening cases the term will be maximal 7 days for a preliminary report with another 8 days for completion of the report.

8.3.2 Annual safety report

In addition to the expedited reporting of SUSARs, the sponsor will submit, once a year throughout the clinical trial, a safety report to the accredited METC, competent authority, Medicine Evaluation Board and competent authorities of the concerned Member States.
This safety report consists of:

- a list of all suspected (unexpected or expected) serious adverse reactions, along with an aggregated summary table of all reported serious adverse reactions, ordered by organ system, per study;
- a report concerning the safety of the subjects, consisting of a complete safety analysis and an evaluation of the balance between the efficacy and the harmfulness of the medicine under investigation.

8.3.3. Pregnancy

Pregnancy is to be strictly avoided during the course of this trial. However, if a female subject becomes pregnant during the study she must be instructed to stop taking the trial medication and immediately inform the investigator. Pregnancies occurring up to 12 weeks after the completion of the trial medication must also be reported to the investigator. The investigator should counsel the subject, discuss the risks of continuing with the pregnancy and the possible effects on the fetus. Monitoring of the patient should continue until conclusion of the pregnancy.

8.4 Follow-up of adverse events

All adverse events will be followed until they have abated, or until a stable situation has been reached. Depending on the event, follow up may require additional tests or medical procedures as indicated, and/or referral to the general physician or a medical specialist.

9 STATISTICAL ANALYSIS

9.1 Descriptive statistics

The main efficacy variable in the comparison of treatments A and B to treatment C (no treatment) is the proportion of subjects in each treatment group achieving loss of HBsAg. The primary objective is to demonstrate superior efficacy of treatment A and/or B to treatment C.

The sample sizes were calculated as follows:

Per treatment group 44 per group (88 for both groups) is needed to achieve 81% power to detect a difference between the two treatment groups and the control group. The proportion in the PEG/IFN and adefovir group is assumed to be 0.01 under the null hypothesis and 0.20 under the alternative hypothesis. The proportion in the control group (no treatment) is 0.01. The statistical test used is the two-sided Fisher's Exact test . The significance level of the test was targeted at 0.05. A group sample of 44 persons in the control group is needed to achieve a 81% power to detect a difference between the PEG/IFN adefovir or PEG/IFN tenofovir.
Regarding a 10% drop-out due to peginterferon side-effects or other reasons a total of 50 patients are needed in each group.

Univariate logistic regression analyses will be performed to identify predictors of response to the combination therapy of PEGASYS® and ADF dipivoxil (Hepsera®) and PEGASYS® and tenofovir. Significant predictors from the univariate analysis will be combined into a multivariate logistic regression analysis. As a rule of thumb, the number of potential predictors to be included in a multivariate analysis should not exceed the number of responders (or non-responders, whichever is the lowest) divided by 10. With 100 patients included, the maximum number of predictors in a multivariate analysis is five, assuming a response rate of 0.50. Initially, the number of significant predictors from univariate analysis to be included in the multivariate analysis will be restricted following multi colinearity diagnostics. If the number of potential predictors remains too large however, a bootstrap procedure will be followed drawing 200 samples of the same size as the original (n=100) with replacement, each time repeating the multivariate logistic regression analysis. Descriptives of this bootstrap procedure will be reported to identify a stable predictor set. The level of significance during all these analyses is set at 0.05.

9.2 .Interim analysis

After inclusion of 50,100 and 150 patients and yearly during longterm follow-up an interim analysis will be performed by the investigator. The statistical test is the two-sided Fisher's exact test. Stopping rules for the study are the occurrence of serious adverse events.

10 ETHICAL CONSIDERATIONS

10.1 Regulation statement

This trial will be conducted in accordance with the current ICH-GCP Guidelines.
Good clinical practice (GCP) is an international ethical and scientific quality standard for designing, conducting, recording, and reporting trials that involve the participation of human subjects. Compliance with these standard provides public assurance that the rights, safety and well being of trial subjects are protected, consistent with the principles that have their origin in the Declaration of Helsinki, and that the clinical trial data are credible.

10.2 Recruitment and consent

Prior to entry in the study, the investigator or a person designated by the investigator must explain to potential subjects or their legally acceptable representative the study and the implications of participation. Subjects will be informed that their participation is voluntary and that they may withdraw from the trial at any time. They will be informed that choosing not to participate or to withdraw from the trial will not have an impact on the care the subject will receive for the treatment of his/her disease. Finally, they will be told that their records may be accessed by the IEC/IRB, regulatory authorities and authorized representatives of the sponsor without violating the confidentiality of the subject, to the extent permitted by the applicable laws and or regulations. By signing the ICF, the subject is authorizing such access.
In case the subject is unable to read and write, an impartial witness must confirm the informed consent.
The subject will be given sufficient time to read the ICF and to ask additional questions. After this explanation and before entry in the trial, consent should be appropriately recorded by means of the subject’s personally dated signature or by the signature of an independent witness who certifies the subject’s consent in writing. After having obtained the consent, a copy or the signed and dated informed consent must be given to the subject.
Any information relevant to the subject’s willingness to participate in the study will be provided to the subject in a timely manner by means of updated ICF. This amended ICF will be signed and dated by the subject and the investigator to document the willingness of the subject to continue with the trial.
This signed and dated amended version will be filled together with initial signed and dated ICF.

10.3 Compensation for injury

The sponsor/investigator has a liability insurance which is in accordance with article 7, subsection 6 of the WMO.

The sponsor (AMR) has an insurance which is in accordance with the legal requirements in the Netherlands (Article 7 WMO and the Measure regarding Compulsory Insurance for Clinical Research in Humans of 23th June 2003). This insurance provides cover for damage to research subjects through injury or death caused by the study.

1. € 450.000,-- (i.e. four hundred and fifty thousand Euro) for death or injury for each subject who participates in the Research;
2. € 3.500.000,-- (i.e. three million five hundred thousand Euro) for death or injury for all subjects who participate in the Research;
3. € 5.000.000,-- (i.e. five million Euro) for the total damage incurred by the organisation for all damage disclosed by scientific research for the Sponsor as ‘verrichter’ in the meaning of said Act in each year of insurance coverage.

The insurance applies to the damage that becomes apparent during the study or within 4 years after the end of the study.

For subject’s participating in this study insurance is covered by the AMR (Academisch Medisch Centrum Medical Research)

10.4 Incentives

Except for travelling expenses no special compensation or incentives will be applicable to participating subjects.

11. ADMINISTRATIVE ASPECTS AND PUBLICATION

11.1 Handling and storage of data and documents

Storage of al study information will be carried out by a database. The outline for this database needs to be specially suited and designed for HBV patients. A person designated by the investigator concerning the development of this database will be applied and will take care of processing this information. Human materials (PBMC's) will be kept in a -80^{0}C freezer until ready for processing. Information concerning this study will only be available by the study investigator and the designated people by the use of passwords.

11.2 Amendments

Amendments are changes made to the research after a favourable opinion by the accredited METC has been given. All amendments will be notified to the METC that gave a favourable opinion.

11.3 Annual progress report

The investigator will submit a summary of the progress of the trial to the accredited METC once a year. Information will be provided on the date of inclusion of the first subject, numbers of subjects included and numbers of subjects that have completed the trial, serious adverse events/ serious adverse reactions, other problems, and amendments.

11.4 End of study report

The investigator will notify the accredited METC of the end of the study within a period of 8 weeks. The end of the study is defined as the last patient's last visit.

In case the study is ended prematurely, the investigator will notify the accredited METC, including the reasons for the premature termination.

Within one year after the end of the study (end of short term follow-up after 72 weeks), the investigator/sponsor will submit a final study report with the results of the study, including any publications/abstracts of the study, to the accredited METC.

11.5 Public disclosure and publication policy

After interim analysis and yearly during long term follow up (5 years) the results of this study will be presented and published.

In accordance with generally recognized principles of scientific collaboration, co-authorship with any company personnel wil be discussed and mutually agreed upon before submission of a manuscript to a publisher.

12. REFERENCES

Reference List

1. World Health Organization. Hepatitis B. World Health Organization Fact Sheet 204 (Revised August 2008). World Health Organization 2007.

2. Lee WM. Hepatitis B virus infection. N Engl J Med 1997 Dec 11;337(24):1733-1745.

3. Yang HI, Lu SN, Liaw YF, You SL, Sun CA, Wang LY, et al. Hepatitis B e antigen and the risk of hepatocellular carcinoma. N Engl J Med 2002 Jul 18;347(3):168-174.

4. Chen YC, Sheen IS, Chu CM, Liaw YF. Prognosis following spontaneous HBsAg seroclearance in chronic hepatitis B patients with or without concurrent infection. Gastroenterology 2002 Oct;123(4):1084-1089.

5. Yuen MF, Wong DK, Sablon E, Tse E, Ng IO, Yuan HJ, et al. HBsAg seroclearance in chronic hepatitis B in the Chinese: virological, histological, and clinical aspects. Hepatology 2004 Jun;39(6):1694-1701.

6. Takkenberg RB, Zaaijer HL, Weegink C.J., Terpstra V, Dijkgraaf MG, Jansen P.L.M., et al. Baseline HBsAg level predict HBsAg loss in HBeAg negative chronic hepatitis B patients treated with a combination of Peginterferon alfa-2a and Adefovir: An interim analysis. J Hepatol 2009 Apr;50(Supp 1):S9-S10.

7. World Health Organization. The World Health Repotrt 1997. 1997.

8. Perz JF, Armstrong GL, Farrington LA, Hutin YJ, Bell BP. The contributions of hepatitis B virus and hepatitis C virus infections to cirrhosis and primary liver cancer worldwide. J Hepatol 2006 Oct;45(4):529-538.

9. Goldstein ST, Zhou F, Hadler SC, Bell BP, Mast EE, Margolis HS. A mathematical model to estimate global hepatitis B disease burden and vaccination impact. Int J Epidemiol 2005 Dec;34(6):1329-1339.

10. World Health Organization. Hepatitis B. World Health Organization Fact Sheet 204 (Revised October 2000). 2007.

11. Lavanchy D. Hepatitis B virus epidemiology, disease burden, treatment, and current and emerging prevention and control measures. J Viral Hepat 2004 Mar;11(2):97-107.

12. Dienstag JL. Hepatitis B virus infection. N Engl J Med 2008 Oct 2;359(14):1486-1500.

13. Lok AS, McMahon BJ. Chronic hepatitis B. Hepatology 2001 Dec;34(6):1225-1241.

14. Dienstag JL, Schiff ER, Wright TL, Perrillo RP, Hann HW, Goodman Z, et al. Lamivudine as initial treatment for chronic hepatitis B in the United States. N Engl J Med 1999 Oct 21;341(17):1256-1263.

15. Marcellin P, Chang TT, Lim SG, Tong MJ, Sievert W, Shiffman ML, et al. Adefovir dipivoxil for the treatment of hepatitis B e antigen-positive chronic hepatitis B. N Engl J Med 2003 Feb 27;348(9):808-816.

16. Hadziyannis SJ, Tassopoulos NC, Heathcote EJ, Chang TT, Kitis G, Rizzetto M, et al. Adefovir dipivoxil for the treatment of hepatitis B e antigen-negative chronic hepatitis B. N Engl J Med 2003 Feb 27;348(9):800-807.

17. Janssen HL, Gerken G, Carreno V, Marcellin P, Naoumov NV, Craxi A, et al. Interferon alfa for chronic hepatitis B infection: increased efficacy of prolonged treatment. The European Concerted Action on Viral Hepatitis (EUROHEP). Hepatology 1999 Jul;30(1):238-243.

18. Wong DK, Cheung AM, O'Rourke K, Naylor CD, Detsky AS, Heathcote J. Effect of alpha-interferon treatment in patients with hepatitis B e antigen-positive chronic hepatitis B. A meta-analysis. Ann Intern Med 1993 Aug 15;119(4):312-323.

19. Tompkins WA. Immunomodulation and therapeutic effects of the oral use of interferon-alpha: mechanism of action. J Interferon Cytokine Res 1999 Aug;19(8):817-828.

20. Kuhen KL, Samuel CE. Mechanism of interferon action: functional characterization of positive and negative regulatory domains that modulate transcriptional activation of the human RNA-dependent protein kinase Pkr promoter. Virology 1999 Feb 1;254(1):182-195.

21. Kuhen KL, Vessey JW, Samuel CE. Mechanism of interferon action: identification of essential positions within the novel 15-base-pair KCS element required for transcriptional activation of the RNA-dependent protein kinase pkr gene. J Virol 1998 Dec;72(12):9934-9939.

22. Franchis de R., Hadengue A, Lau G, Lavanchy D, Lok A, McIntyre N, et al. EASL International Consensus Conference on Hepatitis B. 13-14 September, 2002 Geneva, Switzerland. Consensus statement (long version). J Hepatol 2003;39 Suppl 1:S3-25.

23. Lok AS, Zoulim F, Locarnini S, Bartholomeusz A, Ghany MG, Pawlotsky JM, et al. Antiviral drug-resistant HBV: standardization of nomenclature and assays and recommendations for management. Hepatology 2007 Jul;46(1):254-265.

24. Dienstag JL. Benefits and risks of nucleoside analog therapy for hepatitis B. Hepatology 2009 May;49(5 Suppl):S112-S121.

25. Westland CE, Yang H, Delaney WE, Gibbs CS, Miller MD, Wulfsohn M, et al. Week 48 resistance surveillance in two phase 3 clinical studies of adefovir dipivoxil for chronic hepatitis B. Hepatology 2003 Jul;38(1):96-103.

26. Chu CJ, Hussain M, Lok AS. Quantitative serum HBV DNA levels during different stages of chronic hepatitis B infection. Hepatology 2002 Dec;36(6):1408-1415.

27. Fattovich G, Rugge M, Brollo L, Pontisso P, Noventa F, Guido M, et al. Clinical, virologic and histologic outcome following seroconversion from HBeAg to anti-HBe in chronic hepatitis type B. Hepatology 1986 Mar;6(2):167-172.

28. Hoofnagle JH, Dusheiko GM, Seeff LB, Jones EA, Waggoner JG, Bales ZB. Seroconversion from hepatitis B e antigen to antibody in chronic type B hepatitis. Ann Intern Med 1981 Jun;94(6):744-748.

29. Hoofnagle JH, Doo E, Liang TJ, Fleischer R, Lok AS. Management of hepatitis B: summary of a clinical research workshop. Hepatology 2007 Apr;45(4):1056-1075.

30. Moucari R, Mackiewicz V, Lada O, Ripault MP, Castelnau C, Martinot-Peignoux M, et al. Early serum HBsAg drop: a strong predictor of sustained virological response to pegylated interferon alfa-2a in HBeAg-negative patients. Hepatology 2009 Apr;49(4):1151-1157.

31. Fernandez-Soto L, Gonzalez A, Escobar-Jimenez F, Vazquez R, Ocete E, Olea N, et al. Increased risk of autoimmune thyroid disease in hepatitis C vs hepatitis B before,

during, and after discontinuing interferon therapy. Arch Intern Med 1998 Jul 13;158(13):1445-1448.

32. Marcellin P, Heathcote EJ, Buti M, Gane E, de Man RA, Krastev Z, et al. Tenofovir Disoproxil Fumarate versus Adefovir Dipivoxil for Chronic Hepatitis B. N Engl J Med 2008 Dec 4;359(23):2442-2455.

33. Heathcote EJ, Gane E, de Man RA, Chan S, Sievert W, Mauss S, et al. Two year tenofovir disiproxil fumarate (TDF) treatment and adefovir dipivoxil (ADV) switch data in HBeAg positive patients with chronic hepatitis B (study 103), preliminary results. Hepatology 2008 Oct 1;48(S4):376A.

34. Buster EH, van Erpecum KJ, Schalm SW, Zaaijer HL, Brouwer JT, Gelderblom HC, et al. Treatment of chronic hepatitis B virus infection - Dutch national guidelines. Neth J Med 2008 Jul;66(7):292-306.

35. EMEA summary of product characteristics PEGASYS; 2009.

36. Perry CM, Jarvis B. Spotlight on peginterferon-alpha-2a (40KD) in chronic hepatitis C. BioDrugs 2002;16(3):213-217.

37. Cooksley WG, Piratvisuth T, Lee SD, Mahachai V, Chao YC, Tanwandee T, et al. Peginterferon alpha-2a (40 kDa): an advance in the treatment of hepatitis B e antigen-positive chronic hepatitis B. J Viral Hepat 2003 Jul;10(4):298-305.

38. Marcellin P, Lau GK, Bonino F, Farci P, Hadziyannis S, Jin R, et al. Peginterferon alfa-2a alone, lamivudine alone, and the two in combination in patients with HBeAg-negative chronic hepatitis B. N Engl J Med 2004 Sep 16;351(12):1206-1217.

39. Janssen HL, van ZM, Senturk H, Zeuzem S, Akarca US, Cakaloglu Y, et al. Pegylated interferon alfa-2b alone or in combination with lamivudine for HBeAg-positive chronic hepatitis B: a randomised trial. Lancet 2005 Jan 8;365(9454):123-129.

40. Perrillo R, Hann HW, Mutimer D, Willems B, Leung N, Lee WM, et al. Adefovir dipivoxil added to ongoing lamivudine in chronic hepatitis B with YMDD mutant hepatitis B virus. Gastroenterology 2004 Jan;126(1):81-90.

41. Peters MG, Hann HH, Martin P, Heathcote EJ, Buggisch P, Rubin R, et al. Adefovir dipivoxil alone or in combination with lamivudine in patients with lamivudine-resistant chronic hepatitis B. Gastroenterology 2004 Jan;126(1):91-101.

42. EMEA product information HEPSERA; 2009.

43. Dando T, Plosker G. Adefovir dipivoxil: a review of its use in chronic hepatitis B. Drugs 2003;63(20):2215-2234.

44. Lok AS, McMahon BJ. Chronic hepatitis B. Hepatology 2007 Jan 26;45(2):507-539.

45. EMEA product information VIREAD; 2009.

13. APPENDIX

Figure 1. Natural history of chronic HBV infection

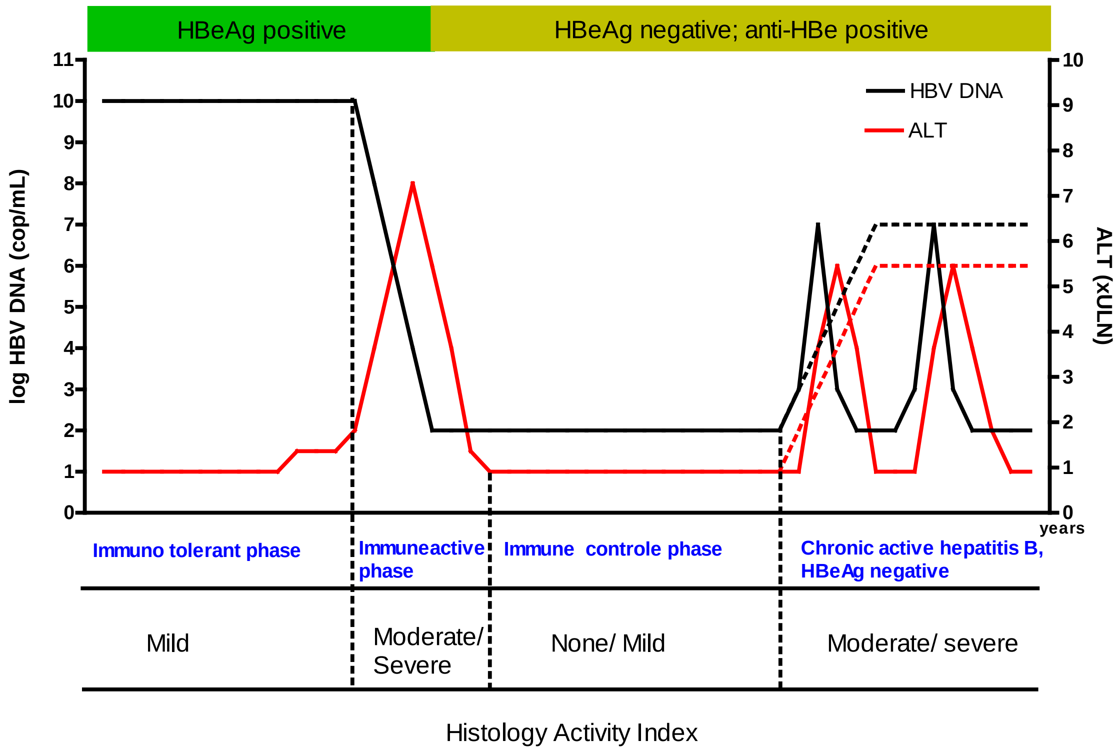

Schedule of Assessments

Table 1. Schedule of Assesments

Assessment/ Procedure	Screening (weeks	Study treatment Period Peg-INF and ADF, Peg-IFN and tenofovir or no treatment (weeks)														Follow-up (weeks)						
Study weeks	**-4 to 0**	**B**	**Day 3**	**1**	**2**	**4**	**6**	**8**	**12**	**18**	**24**	**30**	**36**	**42**	**48**	**2**	**4**	**8**	**12**	**16**	**20**	**24**
Informed consent, medical history	**X**																					
Physical examination	**X**	**X**		**X**	**X**	**X**		**X**	**X**		**X**		**X**		**X**		**X**		**X**			**X**
Liver biopsy (preceding 12 months)	**X**														**X**							
Urine or serum pregnancy test [a]		**X**				X		X	X	X	X	X	X	X	X		X	X	X	X	X	X
Chest X-ray, selected patients [b]	**X**																					
Ultrasound of the liver	**X**														__							
Fibroscan	**X**														**X**							
CT or MRI, selected patients [c]																						
Electrocardiogram, selected patients [d]	**X**																					
Anti-HAV IgM, anti-HIV, anti-HCV, HCV-RNA, anti-HDV	**X**																					
Ophthalmoloic examination [e] (on indication)	**X**																					

**Assessment/ Procedure**	**Long term Follow-up (years)**				
Years	**1**	**2**	**3**	**4**	**5**
Physical examination	**X**	**X**	**X**	**X**	**X**
Complete hematology with differential WBC, and platelets [g] (on indication)					
Chemistry 1 [g]	**X**	**X**	**X**	**X**	**X**
Chemistry 2 [g] (on indication)					
Chemistry 3 (on indication)					
Fibroscan [f]	**X**		**X**		**X**
HBeAg, anti-HBe (frozen for central processing)HBV-DNA (frozen for central processing) [i]HBsAg, anti-HBs (frozen for central processing)	**X**	**X**	**X**	**X**	**X**
Ultrasound, CT or MRI (on indication) c					
PBMC's (frozen for central processing)	**X**	**X**	**X**	**X**	**X**
Paxgene [TM RNA (k)]	**X**	**X**	**X**	**X**	**X**
Plasmatube (proteomics profiling) [(l)]	**X**	**X**	**X**	**X**	**X**
Quality of life [(m)]	**X**	**X**	**X**	**X**	**X**

Table 2 (cont.) Schedule of Assessments

Assessment / Procedure	Screening (weeks)	Study treatment Period (weeks)														Follow-up (week)						
Study weeks	-4 to 0	B	day 3	1	2	4	6	8	12	18	24	30	36	42	48[a]	50[a]	52[a]	56[a]	60[a]	64	68	72[a]
Complete hematology with differential WBC, and platelets [g]	X	X	X	X	X	X	X	X	X	X	X	X	X	X	X	X	X	X	X			X
Chemistry 1 [g]	X	X				X		X	X	X	X	X	X	X	X	X	X	X	X	X	X	X
Chemistry 2 [g]	X	X				X			X		X		X		X				X			X
Chemistry 3	X														X							X
Urinalysis, dipstick (on indication) [g]	X	X				X		X	X		X		X		X		X		X			X
Ceruloplasmin, alfa-1-antitrypsin	X																					
Alfa-fetoprotein	X										X				X							X
AMA, ANA, ASMA, thyroid peroxidase antibodies, selected patients [h]	X														X							
HBeAg, anti-HBe (frozen for central processing)	X	X													X							
HBsAg, anti-HBs (frozen for central processing)	X	X	X	X	X	X	X	X	X	X	X	X	X	X	X	X	X	X	X	X	X	X
HBV-DNA (frozen for central processing) [i]	X	X	X	X	X	X	X	X	X	X	X	X	X	X	X	X	X	X	X	X	X	X
Alpha-2 macro globulin, Haptoglobin, Apolipoprotein A1	X																					
Viral sequencing	X	X	X	X	X	X	X	X	X	X	X	X	X	X	X	X	X	X	X			X

PBMC's (frozen for central processing)	X	X	X	X		X		X	X		X		X		X	X						X
PAXgene[TM] DNA (j) (human genomics)		X																				
PAXgene[TM] RNA [(k)] (human genomics)		X				X			X						X	X						X
Plasma tube (proteomics profiling) [(l)]		X				X			X						X	X						X
Adverse events		X	X	X	X	X	X	X	X	X	X	X	X	X	X	X	X	X	X	X	X	X
Quality of life [(m)]		X							X						X		X					X

Chemistry 1 : ASAT, ALAT,

Chemistry 2: sodium, chloride, potassium, bilirubin, creatinine, urea, prothrombin time, APTT, antitrombine III, alkaline phosphatase, calcium, phosphorus, total protein, albumin, uric acid, TSH, free T_4 and glucose.

Chemistry 3: cholesterol, triglycerides, sober glucose

B: baseline

(a) For females of child bearing potential only, a pregnancy test will be performed within 24 hours prior to first dose. A pregnancy test must be done at any time a secondary amenorrhea of more than 1 week occurs.

(b) Only for patients with pre-existing pulmonary disease (Not necessary if (1) a chest X-ray available to the investigator has been obtained within the past 12 months <u>and</u> (2) the patient's pulmonary disease has been clinically stable)

(c) Patients with cirrhosis or marked fibrosis on liver biopsy or raised AFP need to have a liver imaging study during the screening period to rule out hepatic neoplasia.

(d) For anyone with preexisting cardiac disease.

(e) Eye examination will be done on indication. Any patient complaining of decrease or loss of vision must have a prompt and complete eye examination. Patients with preexisting ophthalmologic disorders (eg, diabetic or hypertensive retinopathy) should receive periodic ophthalmologic exams during Peg-IFN therapy. Peg-IFN treatment should be discontinued in patients who develop new or worsening ophthalmologic disorders.

(f) non-invasive measurent of the liver to detect fibrosis and cirrhosis

(g) Only for patients with decreased kidney function. If there are clinically significant laboratory abnormalities, repeat no less frequently than every 2 weeks or as clinically indicated, with appropriate toxicity management, until they return to normal or baseline values. Urinalysis to be performed via dipstick, with subsequent microscopic evaluation if positive for hemoglobin at the discretion of the investigator.

(h) Only for certain patients at risk, don't repeat if data from previous assessments are available

(i) Roche TaqMan®; when HBV DNA is more then the maximal detection limit, ten or hundred time dilution is proceded.

(j) Paxgene TM DNA sample (tube) will be collected. The DNA samples will be used exclusively for exploratory DNA research (genotyping of hostgenomics) to evaluate drug disposition genes.(e.g. metabolic enzymes and drug transporters), to expore possible underlying genetic variants involved in therapy response in HBV chronically infected.Samples will only be processed in a number of subjects. No other testing will be performed on these samples.

(k) Paxgene TM RNA sample (tube) will be collected to assess the expression of RNA in peripheral blood using whole genome microarray technology. These samples ares collected as part of an effort to better understand the effects of medication. Samples will only be processed in a number of subjects. No other testing will be performed on these samples.

(l) plasma tube will be collected for proteomics profiling. These samples are collected as part of a study to better understand the effects of medication on liver activity. Samples will only be processed in a number of subjects. No other testing will be performed on these samples. Possible protein candidates include: SR-B1(CD36), CD81, certain Claudins, STAT-1 and 2, ApoE and more.

(m) During the study a quality of life index according to SF36 standardized questionnaires will be administered

Screening Assessments

Table 3. Screening Assessments

Medical History And Physical Examination	Includes body weight, vital signs
Eye examination	Eye examination by an ophthalmologist at screening will be done on indication.Patients with preexisting ophthalmologic disorders (eg, diabetic or hypertensive retinopathy) should receive periodic ophthalmologic exams during Peg-IFN therapy. Peg-IFN treatment should be discontinued in patients who develop new or worsening ophthalmologic disorders.
Clinical Chemistry	ASAT, ALAT, bilirubin, creatinine, urea, sodium, chloride, potassium, calcium, phosphorus, prothrombin time, alkaline phosphatase, total protein, albumin, BUN, uric acid, cholesterol, triglycerides, glucose
Hematology	Leukocyte count, differential WBC, red blood count, platelets
Immunology and Special Chemistry	HBeAg, anti-HBe, HBsAg, anti-HBs<br>anti-HIV, anti-HCV, anti-HDV, Alfa-fetoprotein, ceruloplasmin, alfa1-antitrypsin, ferritin<br>Only for patients at risk without previous data being available: AMA, ANA, ASMA, thyroid peroxidase antibodies.
Virology	Quantitative HBV-DNA measurement
Thyroid Function Tests	TSH, free T_4
Urinalysis	Dipstick with subsequent microscopic evaluation if positive for hemoglobin
Chest X-Ray	Will be done during screening (Not necessary if (1) a chest X-ray available to the investigator has been obtained within the past 12 months and (2) the patient's pulmonary disease has remained clinically stable.)
Electrocardiogram	Only for anyone with a history of pre-existing cardiac disease.
Liver Biopsy [(a)]	Performed at start of the study and repeated after 48 weeks.
Fibroscan [(b)]	Performed at start of the study and repeated after 48 weeks.
Liver Imaging (ultrasound)	Performed at screening or start of the study, repeated after 48 weeks and on indication.
Liver Imaging (CT or MRI)	Only for patients suspected to have hepatic neoplasia.
HCG Pregnancy Test	For women of childbearing potential a negative urine (or serum) HCG test needs to be documented within 24 hours prior to the first dose.

(a) When a participant has objections against a second liver biopsy at week 48 ,he or she can refuse it. The participant will than not be excluded from the study.

(b) A fibroscan measures the liver stiffness, expressed in kilopascals (kPA)
