# Supplementary material for: A Prospective Five-Year Follow-up After peg-Interferon Plus Nucleotide Analogue Treatment or no Treatment in HBeAg Negative Chronic Hepatitis B Patients
Source: J Clin Exp Hepatol. 2022 Jan 4;12(3):735–44. doi: 10.1016/j.jceh.2021.12.011 (PMC9168707; doi:10.1016/j.jceh.2021.12.011)
Supplement: Supplementary data [file mmc3.docx]

***SUPPLEMENTARY***

A prospective 5-year follow-up after Peg-interferon plus nucleotide analogue treatment or no treatment in HBeAg negative chronic hepatitis B patients

***Authors***  *R. Erken MD, V.V. Loukachov MD,* *A. de Niet MD PhD, L. Jansen MD PhD, F. Stelma MD PhD, J.T. Helder MSc, M.W. Peters, H.L. Zaaijer MD PhD, N.A. Kootstra PhD, S.B. Willemse MD PhD, H.W. Reesink MD PhD.*

***Supplementary tables***

- - *Table 1, Baseline difference between patients achieving and not achieving HBsAg-loss*
  - *Table 2, Proportions of HBsAg-loss between patients with genotype A and other genotypes.*
  - *Table 3 Proportions of HBsAg-loss between HBeAg negative patients in the chronic infection phase and patients that did not meet these criteria at baseline.*

***Supplementary figures***

- - *Figure 1, Dynamics virology markers divided by treated and untreated patients that achieve or did not achieve HBsAg-loss during study or follow-up.*
  - *Figure 2. HBsAg changes over time for patients with HBV genotype A versus other genotypes.*
  - *Figure 3. ROC curve analysis*
  - *Figure 4A-D. Virology dynamics for patients meeting or not meeting the ‘HBeAg negative chronic infection’ phase criteria.*
  - *Figure 5A-B. Dynamics of fibrosis score (A) and ALT (B) levels over time for each allocation arm.*

**Supplementary Table 1**

|  | *Peg-IFN plus adefovir  (n=43)* | | *Peg-IFN plus tenofovir  (n=34)* | | *No treatment*  *(n=41)* | |
| --- | --- | --- | --- | --- | --- | --- |
| *HBsAg-loss* | *Yes   (n=8)* | *No*  *(n=35)* | *Yes*  *(n=4)* | *No*  *(n=30)* | *Yes*  *(n=6)* | *No*  *(n=35)* |
| Age, years | 43 (11)* | 53 (11)* | 48 (4) | 44 (12) | 47 (7) | 42 (10) |
| Female sex | 3 (37·5) | 14 (40·0) | 2 (50·0) | 17 (56·7) | 1 (6·7) | 15 (42·9) |
| Ethnicity  Caucasian  Asian  African  South American | 2 (25·0)  3 (37·5)  2 (25·0)  1 (12·5) | 8 (22·9)  15 (42·9)  5 (14·3)  7 (20·0) | 1 (25·0)  0 (0·0)  2 (50·0)  1 (25·0) | 10 (33·3)  7 (23·3)  6 (20·0)  7 (23·3) | 2 (5·7)  2 (5·7)  2 (5·7)  0 (0·0) | 14 (40·0)  11 (31·4)  5 (14·3)  5 (14·3) |
| ALT (U/L) | 22 (19-23)* | 30 (22-45)* | 23 (15-69) | 26 (19-30) | 37 (13-38) | 36 (21-49) |
| Peg-IFN naive | 8 (100) | 32 (91·4) | 3 (75·0) | 28 (93·3) | 6 (100) | 35 (100) |
| HBV genotype  A  B  C  D  E  G  Indeterminable | 3 (37·5)  1 (16·7)  0 (0·0)  0 (0·0)  2 (25·0)  0 (0·0)  2 (25·0) | 7 (20·0)  3 (8·6)  2 (5·7)  10 (28·6)  7 (20·0)  0 (0·0)  6 (17·1) | 2 (50·0)  1 (25·0)  0 (0·0)  1 (25·0)  0 (0·0)  0 (0·0)  0 (0·0) | 5 (16·7)  2 (6·7)  1 (3·3)  10 (33·3)  4 (13·3)  0 (0·0)  8 (26·7) | 2 (33·3)  0 (0·0)  0 (0·0)  2 (33·3)  1 (16·7)  0 (0·0)  1 (16·7) | 6 (17·1)  2 (5·7)  3 (8·6)  9 (25·7)  7 (20·0)  1 (2·9)  7 (20·0) |
| HBsAg (log_10_ IU/mL) | 1·98 (1·42)** | 3·46 (0·63)** | 2·63 (0·93)* | 3·53 (0·54)* | 2·16 (0·99)* | 3·18 (0·79)* |
| HBV-DNA (log_10_ IU/mL) | 2·32 (1·63) | 2·71 (1·19) | 1·83 (1·03)* | 2·94 (1·01)* | 2·18 (1·07) | 2·86 (1·04) |
| HBV-DNA <2000 IU/mL, yes | 6 (75·0) | 24 (68·6) | 4 (100) | 16 (53·3) | 5 (83·3) | 24 (68·6) |
| Fibroscan kPa | 4·6 (1·1) | 5·1 (1·9) | 4·8 (1·5) | 5·4 (1·8) | 6·9 (3·6) | 5·7 (1·5) |
| Liver biopsies done | 7 (87·5) | 28 (80·0) | 2 (50·0) | 26 (86·7) | 5 (83·3) | 14 (40) |
| Ishak fibrosis score  0  1  >1 | 2 (28·6)  4 (57·1)  1 (14·3) | 5 (17·9)  19 (67·9)  4 (14·3) | 0 (0·0)  2 (100)  0 (0·0) | 3 (11·5)  15 (57·7)  8 (30·8) | 3 (60·0)  2 (40·0)*  0 (0·0) | 1 (7·1)  12 (85·7)*  1 (7·1) |
| Steatosis grade  0  1  ≥ 2  Unknown | 4 (57·1)  1 (14·3)  2 (28·6)  0 (0·0) | 18 (64·3)  9 (32·1)  1 (3·6)  0 (0·0) | 1 (50·0)  1 (50·0)  0 (0·0)  0 (0·0) | 16 (61·5)  7 (26·9)  2 (7·7)  1 (3· 8) | 2 (40)*  2 (40)  1 (20)  0 (0·0) | 11 (78·6)*  0 (0·0)  3 (21·4)  0 (0·0) |

**Baseline difference between patients achieving and not achieving HBsAg-loss.** *p-value <0·05. **p-value <0·001 comparing patients with and without HBsAg-loss within the same treatment arm using X^2^-test, Mann Whitney-U or students t-test where applicable. Abbreviations: ALT, alanine aminotransferase; HBV, hepatitis B virus; HBeAg, hepatitis B e antigen; HBsAg, hepatitis B surface antigen; Peg-IFN, peg-interferon-alfa-2a;

**Supplementary Table 2**

|  | Proportion HBsAg-loss | | Compare genotype  A vs other |
| --- | --- | --- | --- |
| Allocation | **Genotype A** | **Genotype other** | **p-value** |
| Peg-IFN plus adefovir (n=43) | 3/10 (30%) | 5/33 (15%) | 0.362 |
| Peg-IFN plus tenofovir (n=34) | 2/7 (29%) | 2/27 (7%) | 0.180 |
| No treatment (n=41) | 2/8 (25%) | 4/33 (12%) | 0.578 |

**Proportion of patients achieving HBsAg-loss at 5-years after treatment shown separate for patients with genotype A and patients with another or indeterminable genotype.**  Within each allocated arm, proportions of patients achieving HBsAg-loss were compared between patients with genotype A and other patients using an X^2^-test. Abbreviations: HBsAg, hepatitis B surface antigen; Peg-IFN, peg-interferon-alfa-2a.

**Supplementary Table 3**

| HBsAg-loss | Treated | Untreated |
| --- | --- | --- |
| HBeAg negative chronic infection | 10/62 (16%) | 4/26 (15%) |
| HBeAg negative,  ALT > ULN or HBV DNA > 2,000 | 2/15 (13%) | 2/15 (13%) |

**Proportions of HBsAg-loss between HBeAg negative patients in the chronic infection phase and patients that did not meet these criteria at baseline.** Patients treated with Peg-IFN and tenofovir or adefovir were combined. None of the proportions differed (p>0·735) between groups using an X^2^-test. Abbreviations: ALT, alanine aminotransferase; HBV, hepatitis B virus; HBsAg, HBeAg, hepatitis B e antigen; hepatitis B surface antigen; Peg-IFN, peg-interferon-alfa-2a.

**Supplementary figure 1A-B**

**A**

**B**

**Dynamics virology markers divided by treated and untreated patients that achieve or did not achieve HBsAg-loss during study or follow-up.** HBsAg (A) and HBV DNA (B) levels over time, standard deviation. HBsAg, hepatitis B surface antigen; HBV, hepatitis B virus.

**Supplementary Figure 2**

**B**

**C**

**A**


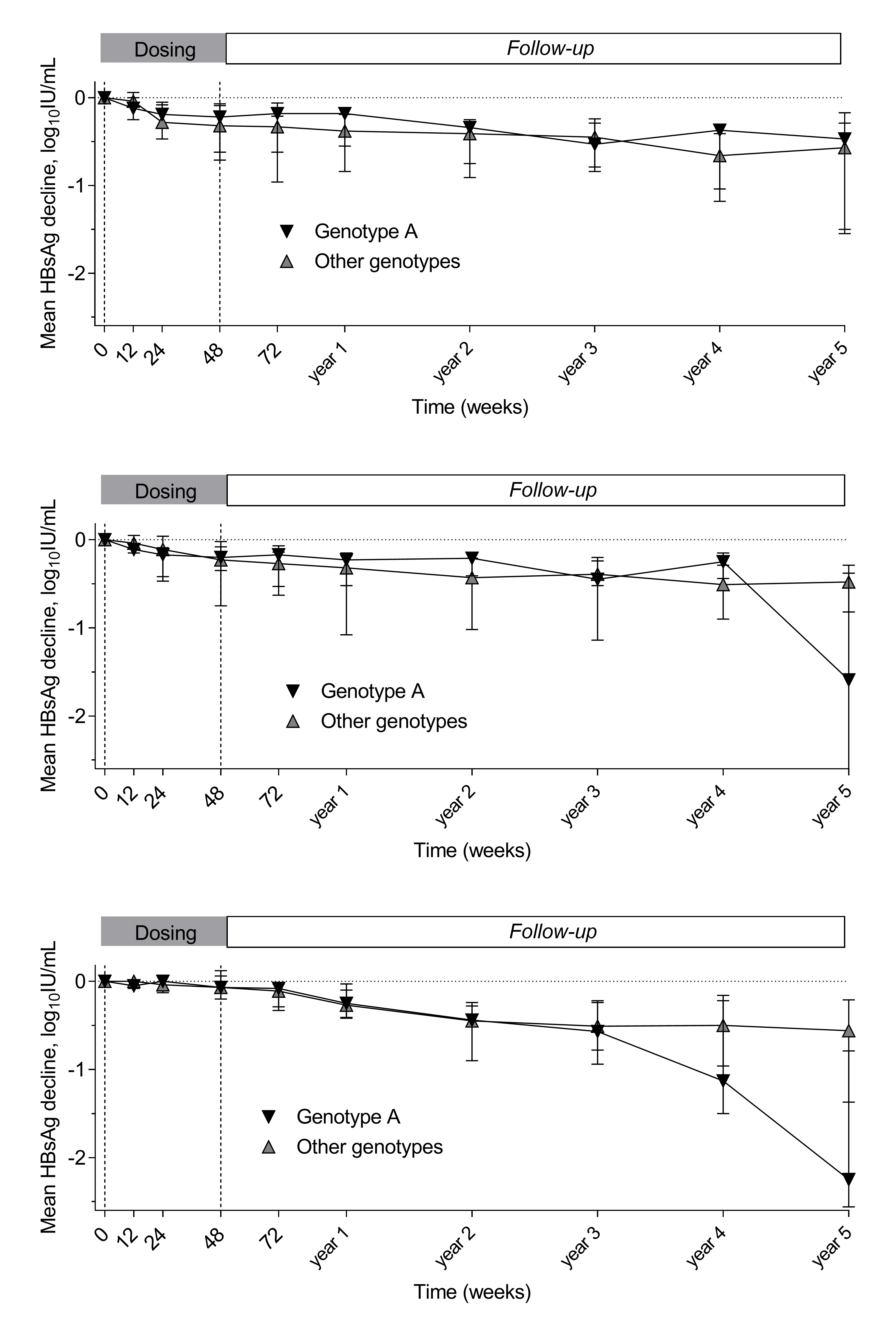


**HBsAg dynamics for patients with Genotype A versus patients with another of indeterminable genotype**. Median change from baseline for HBsAg levels in the Peg-IFN plus adefovir (A) Peg-IFN plus tenofovir (B) and untreated (C) group. Symbols; ▼ black downward triangle, patients with a HBV Genotype A infection, ▲ upward grey triangle, patients with a other HBV Genotype infection; error bars, interquartile range. Abbreviations: HBsAg, hepatitis B surface antigen; HBV, hepatitis B virus*.*

**Supplementary Figure 3**


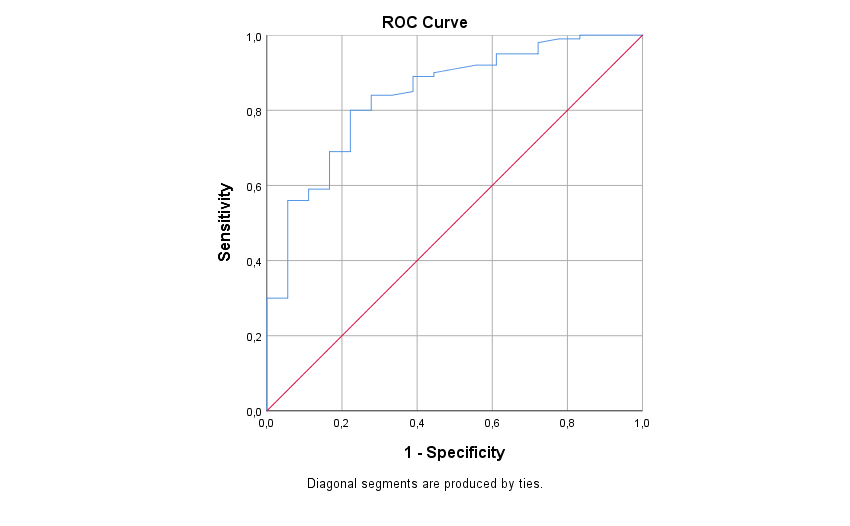


**ROC curve analysis** on baseline HBsAg levels (Log_10_ IU/mL) predicting functional cure during treatment and 5 year follow-up, with an area under the curve of 0·841. Abbreviations: ROC, relative operating characteristic.

**Supplementary Figure 4A-D**

**Virology dynamics for patients meeting or not meeting the ‘HBeAg negative chronic infection’ phase criteria**. Mean Change from baseline for HBsAg levels in the treatment group (A) and the untreated group (B). Change form baseline for HBV DNA levels in the treatment group (C) and the untreated group (D). Symbols; ▼ black downward triangle, patients in the ‘grey zone’ not meeting the criteria for HBeAg negative chronic infection; **○** circle, patients meeting the criteria for HBeAg negative chronic infection; error bars, standard error of the mean. Abbreviations: HBsAg, hepatitis B surface antigen; HBV, hepatitis B virus*.*

**Supplementary Figure 5A-B**

**A**

******

******

**B**

*****

*****

**Dynamics of fibrosis score (A) and ALT (B) levels over time for each allocation arm.** Symbols; ▲ black upward triangle, Peg-IFN plus tenofovir; ■ black square, untreated patients; **○** open circle, Peg-IFN plus adefovir; error bars, standard error of the mean. Abbreviations: ALT, alaninetransferase; EOT, end of treatment; kPa, kilopascal. * p<0.05, significantly lower ALT levels in no-treatment group compared to both treatment group separately, ** p<0.05, significantly lower fibrosis level by means of fibroscan in the Peg-IFN plus adefovir group.
